# Normal transcription of cellulolytic enzyme genes relies on the balance between the methylation of H3K36 and H3K4 in *Penicillium oxalicum*

**DOI:** 10.1186/s13068-019-1539-z

**Published:** 2019-08-20

**Authors:** Yanan Li, Yueyan Hu, Zhu Zhu, Kaili Zhao, Guodong Liu, Lushan Wang, Yinbo Qu, Jian Zhao, Yuqi Qin

**Affiliations:** 1 National Glycoengineering Research Center, Shandong University, Qingdao, 266237 China; 2 State Key Lab of Microbial Technology, Shandong University, Qingdao, 266237 China

**Keywords:** Cellulolytic enzymes, *Penicillium oxalicum*, H3K4 methylation, H3K36 methylation

## Abstract

**Background:**

Enzymatic hydrolysis of lignocellulose by fungi is a key step in global carbon cycle and biomass utilization. Cellulolytic enzyme production is tightly controlled at a transcriptional level. Here, we investigated the roles of different histone lysine methylation modifications in regulating cellulolytic enzyme gene expression, as histone lysine methylation is an important process of chromatin regulation associated with gene transcription.

**Results:**

*Po*Set1 and *Po*Set2 in *Penicillium oxalicum*, orthologs of Set1 and Set2 in budding yeast, were associated with the methylation of histone H3 lysine 4 (H3K4) and lysine 36 (H3K36). Cellulolytic enzyme production was extensively upregulated by the disruption of *Po*Set2, but was significantly downregulated by the disruption of *Po*Set1. We revealed that the activation of cellulolytic enzyme genes was accompanied by the increase of H3K4me3 signal, as well as the decrease of H3K36me1 and H3K36me3 signal on specific gene loci. The repression of cellulolytic enzyme genes was accompanied by the absence of global H3K4me1 and H3K4me2. An increase in the H3K4me3 signal by *Poset2* disruption was eliminated by the further disruption of *Poset1* and accompanied by the repressed cellulolytic enzyme genes. The active or repressed genes were not always associated with transcription factors.

**Conclusion:**

H3K4 methylation is an active marker of cellulolytic enzyme production, whereas H3K36 methylation is a marker of repression. A crosstalk occurs between H3K36 and H3K4 methylation, and *Po*Set2 negatively regulates cellulolytic enzyme production by antagonizing the *Po*Set1-H3K4me3 pathway. The balance of H3K4 and H3K36 methylation is required for the normal transcription of cellulolytic enzyme genes. These results extend our previous understanding that cellulolytic enzyme gene transcription is primarily controlled by transcription factors.

**Electronic supplementary material:**

The online version of this article (10.1186/s13068-019-1539-z) contains supplementary material, which is available to authorized users.

## Introduction

In the global carbon cycle, nearly 1.6 × 10^10^ tons of carbon is fixed by photosynthetic terrestrial plants every year, but less than 3% is currently utilized. Lignocellulose is the main component of plant cell wall and the most abundant biomass in the world. The microorganismal enzymatic hydrolysis of lignocellulose is a key step in global carbon cycle and biomass utilization. Cellulolytic enzymes secreted by numerous fungi, such as *Trichoderma*, *Aspergillus*, *Neurospora*, *Penicillium*, *Fusarium*, and *Magnaporthe*, break down lignocellulose to small molecules, helping to promote the utilization of biomass and completing the carbon cycle in nature [1, 2]. Cellulolytic enzymes are also important in many industries, particularly in textile and paper industries and in cellulosic ethanol production.

Cellulolytic enzyme production in fungi is tightly controlled at a transcriptional level [3]. Transcription factors (TFs), such as Cre1/CreA, Xyr1/XlnR, and Clr2/ClrB, play important roles in cellulolytic gene transcription regulation [4–6]. In addition to the key TFs, chromatin regulation is reported to be associated with the transcriptional activation/repression of genes that encode cellulolytic enzymes [7–9], although the regulation mechanism has yet to be elucidated. In eukaryotic cells, chromatin is not static but is highly dynamic. Chromatin can be altered by enzymes that have histone-modifying activities or ATP-dependent nucleosome-remodeling activities [10, 11]. Such modifications affect accessibility of the DNA, and therefore affect processes such as replication, repair, recombination, and transcription [12, 13].

Histone lysine methylation is an important part of histone modification that is widely associated with a range of key cellular processes [14, 15]. In the budding yeast *Saccharomyces cerevisiae*, three well-studied methylated lysine residues are present, namely, histone H3 lysine 4 (H3K4), histone H3 lysine 36 (H3K36), and histone H3 lysine 79 (H3K79). *S. cerevisiae* histone methyltransferase Set1 and Set2, possessing the evolutionarily conserved Su(var)3-9, Enhancer-of-zeste, and Trithorax (SET) domain, perform H3K4 and H3K36 methylation, respectively [16]. H3K4 methylation is generally associated with active and euchromatic domains. In *S. cerevisiae*, H3K4me3 localizes to the 5′ end of active genes and is associated with the RNA polymerase II (Pol II) complex during transcription initiation [17]. Set2 is related to the hyperphosphorylated carboxyl-terminal domain of RNA Pol II and deposits the trimethyl group onto H3K36 during transcriptional elongation [18]. H3K36me3 recruits various effector proteins and complexes, such as Ioc4 [19] and NuA3 acetyltransferase complex [20], which coordinate transcriptional elongation at coding regions. In addition to H3K4, H3K36, and H3K79, histone H3 lysine 9 (H3K9) and histone H3 lysine 27 (H3K27) participate in transcriptional repression [21, 22].

A small number of studies have described the roles of H3K4 or H3K36 methylation in regulating growth, pathogenicity, and secondary metabolism of filamentous fungi [23–26]. Although cellulolytic enzymes are typical glycoside hydrolases, which are prominent extracellular proteins produced by diverse fungi, the role of histone methylation in the regulation of cellulolytic enzyme genes is poorly elucidated. A putative methyltransferase Lae1 is a positive regulator of the transcription of cellulolytic genes in *Trichoderma reesei* [27], but its histone target has yet to be determined [28]. As such, whether and how different histone methylation modifications affect the transcription of cellulolytic genes remain unclear.

*Penicillium oxalicum* possesses various cellulolytic enzymes, including typical cellulase, hemicellulase, and other carbohydrate-active enzymes, as well as cellulolytic enzyme-related regulators [29], which are conserved in many other cellulolytic enzyme-producing filamentous fungi, such as *T. reesei*, *Aspergillus niger*, and *Neurospora crassa*. *P. oxalicum* has been established as a model system to elucidate the regulatory mechanism of cellulolytic enzyme gene expression. In the present study, we found that the balance of H3K4 and H3K36 methylation is required for the normal transcription of cellulolytic enzyme genes. We demonstrated for the first time that a crosstalk occurs between H3K36 and H3K4 methylation, and *Po*Set2 negatively regulates cellulolytic enzyme production by antagonizing the *Po*Set1-H3K4me3 pathway.

## Results

### Disruption of *Poset2* resulted in developmental defects and high cellulolytic enzyme secretion

**Fig. 1:**
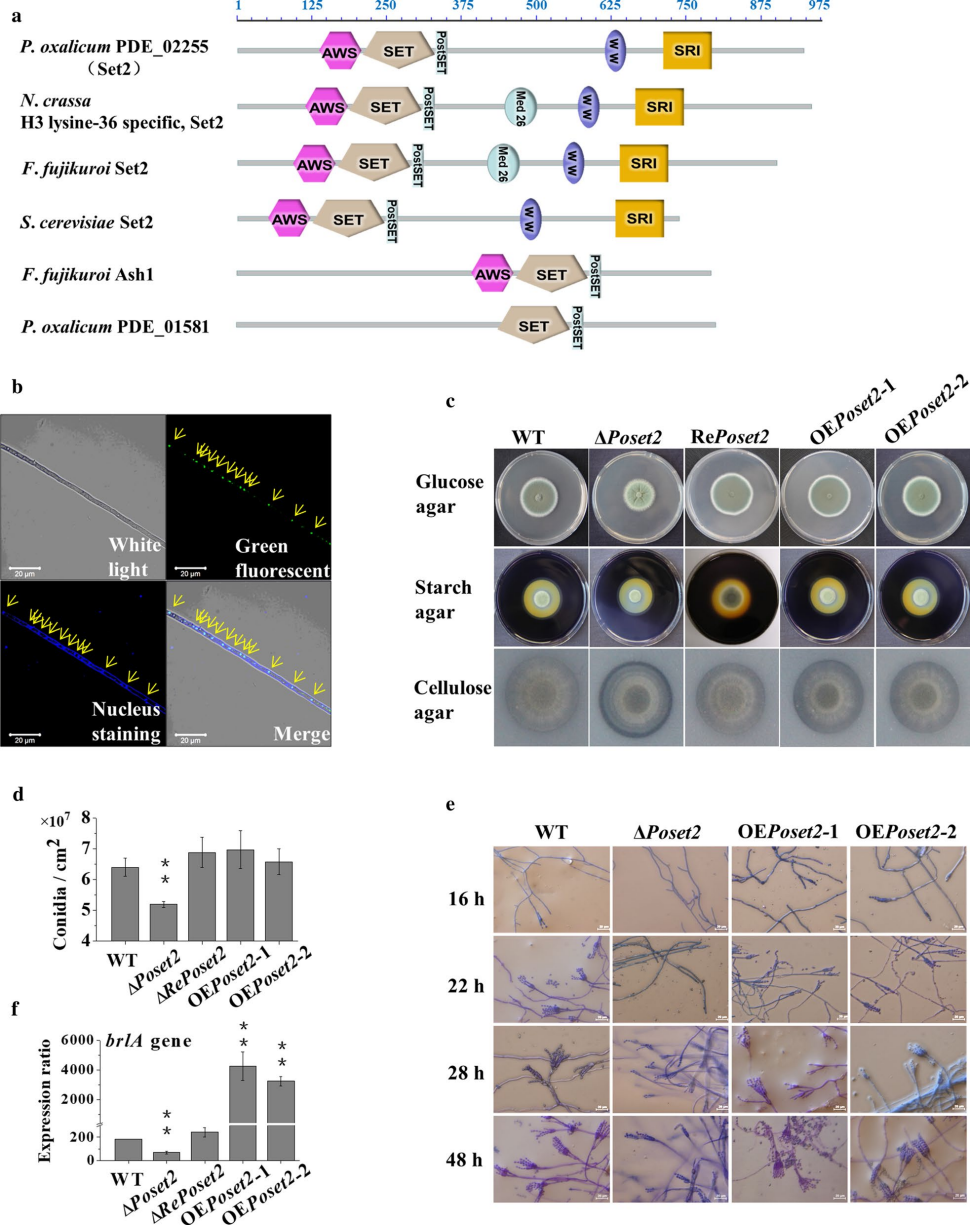
Phenotype analysis of *Po*Set2 mutants. **a** Domain architecture analysis of *Po*Set2 orthologs. Referring to the websites SMART (http://smart.embl-heidelberg.de/) and Pfam database (http://pfam.xfam.org/), the maps were constructed with equal proportions of the respective sequences. A diagram of five conserved domains of Set2 orthologs: AWS, SET, PS, WW, and SRI domains. **b** Subcellular localization of *Po*Set2 in *P. oxalicum*. Upper left, white light; upper right, green fluorescence (green dots); bottom left, nuclear staining (blue dots); bottom right, merged (white dots). Yellow arrows indicating the location that showed green fluorescence and nuclear staining and the overlap of green dots and blue dots on the merged image (white dots). **c** Morphological characteristics of the colony on Vogel’s agar with 2% glucose, 1% starch, and 0.5% ball-milled microcrystalline cellulose. 1.5 μL of fresh spore solutions (10^7^/mL) were spotted on Vogel’s medium ager plates with different carbon source, and then cultivated at 30 °C for 3–5 days. **d** Levels of conidiation on Vogel’s agar with 2% glucose. **e** Observation of mycelial growth and sporogenesis of WT and *Po*Set2 mutants. The length of the size bar shown in the figure represented an actual length of 20 μm. **f** Assay of the transcription level of *brlA*. Statistical significance tests were performed by one tailed, unequal variance *t*-test. *P < 0.05, **P < 0.01, ***P < 0.001

PDE_02255 (GenBank EPS27312.1), an ortholog of *S. cerevisiae* Set2, was identified within the *P. oxalicum* genome. The putative amino acid sequence of PDE_02255 shared 45% identity with the sequence of yeast Set2. Phylogenetic analysis indicated that PDE_02255 was similar to *Fusarium fujikuroi* Set2 and *N. crassa* Set2 (Additional file 1: Figure S1). PDE_02255 contains an Associated With SET (AWS), SET, a post-SET (PS) domain; a WW domain; and a Set2–Rpb1 interaction (SRI) domain (Fig. 1a). All of them were highly conserved domain architectures found in typical Set2-related enzymes [26]. Hereafter, PDE_02255 was named *Po*Set2.

We fused the *Poset2*-coding sequence with *GFP* and introduced it to *P. oxalicum* to obtain the strain *Po*Set2–GFP. The overlap of green fluorescence and nuclear staining on the merged image (Fig. 1b, bottom right, yellow arrows) indicated the nuclear localization of *Po*Set2 in *P. oxalicum*. The deletion of *Poset2* (Δ*Poset2*) led to asexual developmental defects. The Δ*Poset2* colony was wrinkled in the center and had a light bean green color compared with the dark-green WT when the strains were cultivated on glucose agar, suggesting that *Poset2* deletion impaired asexual spore formation (Fig. 1c). Conidiation in the Δ*Poset2* mutant was reduced by ~ 25.0% of that in the WT after 5 days of cultivation on glucose (Fig. 1d). WT started to produce premature conidia after 16 h of cultivation, and the mature conidia with characteristic brush-like structures formed after 22 h. Δ*Poset2* delayed the initiation of asexual development as the conidia did not form until the 28th h, and a low amount of phialide was formed. When the cultivation time was prolonged to 48 h, the Δ*Poset2* mutant still showed a low amount of phialide (Fig. 1e). The re-complement strain (Re*Poset2*) and the two *Poset2* overexpression strains (i.e., OE*Poset2*-1 and OE*Poset2*-2), which were randomly selected, had a phenotype identical to that of WT (Fig. 1). The classical genetic model of conidiation in important ascomycetous fungi, such as *Aspergillus* and *Penicillium*, has indicated that the transcription factor BrlA, as the first regulator in the major regulatory pathway of conidiation, is necessary to direct conidiation [30]. *brlA* expression is required for conidiation in *P. oxalicum* [31]. We observed a downregulated transcription level of *brlA* in Δ*Poset2* (Fig. 1f), suggesting that *Po*Set2 was involved in conidiation through the regulation of the BrlA regulatory pathway. Furthermore, we noticed a clearer cellulolytic halo around the Δ*Poset2* colony compared with that in WT when the strains were grown on Vogel’s medium plus cellulose agar, implying a higher cellulolytic enzyme secretion in Δ*Poset2* than in WT (Fig. 1c).

### Extensive upregulation of genes that encode cellulolytic enzymes was observed in Δ*Poset2*

Upon the observation of a clear cellulolytic halo around the Δ*Poset2* colony, we explored whether the improvement of cellulolytic enzyme secretion was due to the upregulated transcription of the corresponding genes. We performed a genome-wide expression profiling analysis between WT and Δ*Poset2* to obtain a global view of the role of *Po*Set2 in regulating gene expression. With the number of sequenced reads increasing, the number of identified genes was also increased. When the number of sequenced reads reached a certain amount, the growth curve of identified genes flattened, indicating that the number of identified genes tended to reach the saturation. For each sample, > 10 million reads of mRNAs were obtained. The results of saturation analysis indicated that the depth of sequencing data was sufficient for information analysis (Additional file 2: Figure S2).

**Fig. 2:**
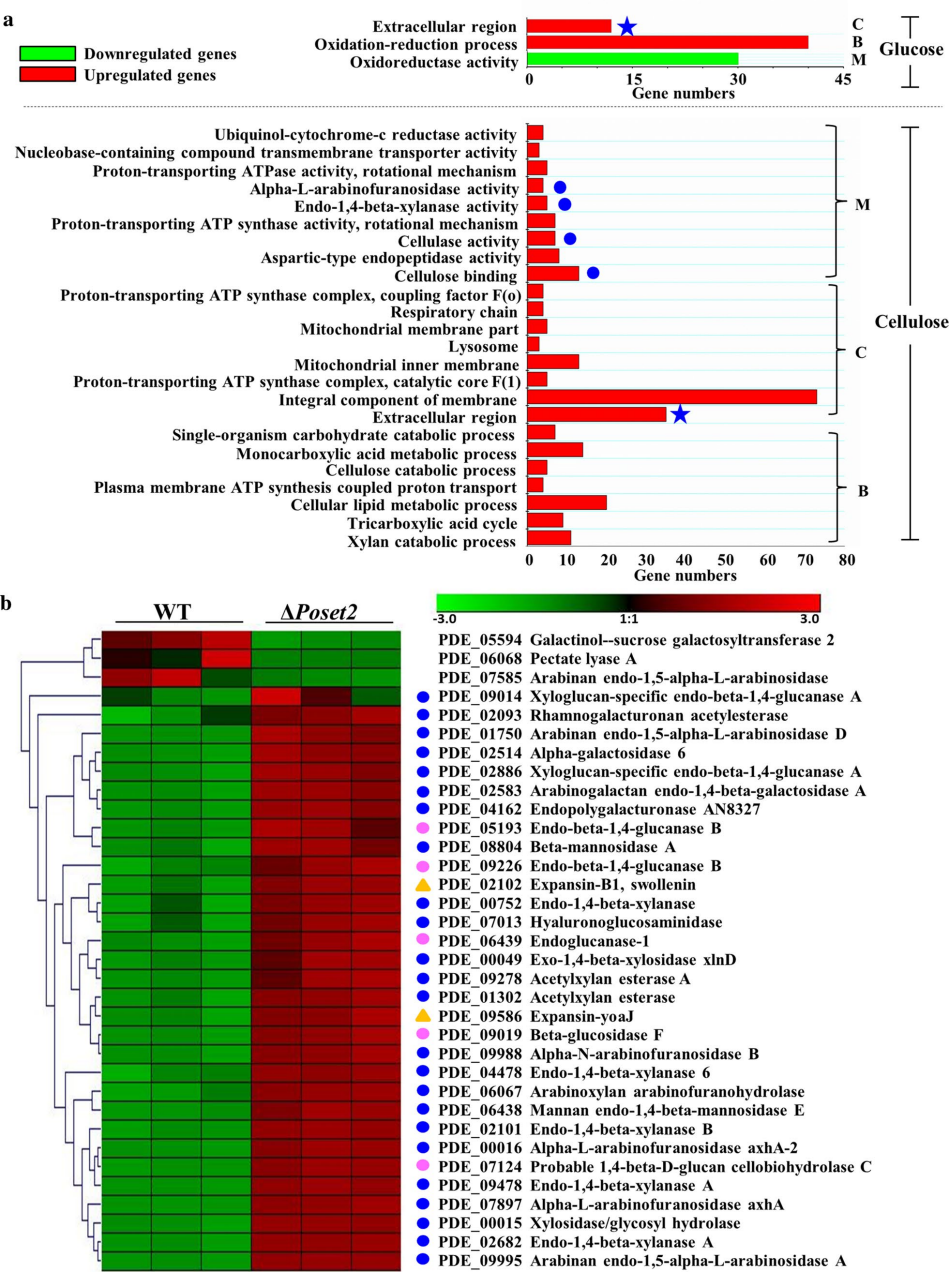
Function enrichment and Clustering analysis. **a** Function enrichment of the upregulated or downregulated (≥ twofold, probability ≥ 0.8) genes in Δ*Poset2* compared with that of WT after the strains were cultivated in the glucose medium (up) or in the cellulose medium (down). The function enrichment analysis was conducted in Blast2GO [68]. B, C, and M indicate the GO category. B, biological process; C, cellular component; M, molecular function. Green bars, function enrichment analysis of downregulated genes; red bars, function enrichment analysis of the upregulated genes. Blue stars indicate the same category of enrichment in both media. **b** Clustering analysis of genes encoding the secreted proteins by using Genesis. The gradient color bar code at the top indicates a value at log2 fold change of expression in the treatment case to the expression in the control case. Blue dots, genes encoding hemicellulase; pink dots, genes encoding cellulose; yellow triangle, genes encoding others

When the strains were cultivated in the glucose medium, which is a repression medium for cellulolytic enzyme formation, significant expression differences (fold change ≥ 2, probability ≥ 0.8) in 469 gene expression were observed in Δ*Poset2* compared with that in WT (Additional file 3: Spreadsheet S1). Among the regulated genes, 276 genes (58.8%) mainly involved in the extracellular region (GO category: cellular component) and the oxidation–reduction process (GO category: biological process) were upregulated in Δ*Poset2* (Fig. 2a, up, red bars). Among the regulated genes, 193 genes (41.2%) mainly implicated in the oxidoreductase activity (GO category: molecular function) were downregulated (Fig. 2a, up, green bar).

When the strains were cultivated in the cellulose medium, which is an inducing medium for cellulolytic enzyme formation, significant expression differences (fold change ≥ 2, probability ≥ 0.8) in 1457 genes were observed in Δ*Poset2* compared with that of WT (Additional file 4: Spreadsheet S2). Among the regulated genes, 982 genes (67.4%) were downregulated in Δ*Poset2*, but no enrichment was obtained via GO analysis. A total of 475 genes (32.6%) were upregulated in Δ*Poset2* (Fig. 2a, down, red bars), which were mainly involved in ubiquinol–cytochrome-*c* reductase activity, nucleobase-containing compound transmembrane transporter activity, proton-transporting ATPase activity, α-l-arabinofuranosidase activity, endo-1,4-β-xylanase activity, proton-transporting ATP synthase activity, cellulase activity, aspartic-type endopeptidase activity, cellulose binding (GO category: molecular function), in proton-transporting ATP synthase complex coupling factor F(o), respiratory chain, mitochondrial membrane part, lysosome, mitochondrial inner membrane, proton-transporting ATP synthase complex catalytic core F(1), integral component of membrane, extracellular region (GO category: cellular component), and in single-organism carbohydrate catabolic process, monocarboxylic acid metabolic process, cellulose catabolic process, plasma membrane ATP synthesis coupled proton transport, cellular lipid metabolic process, tricarboxylic acid cycle, xylan catabolic process (GO category: biological process) (Fig. 2a, up, red bars). The predicted functions of the upregulated genes under the molecular function GO category are listed in Additional file 5: Table S1. Specifically, we noticed the enrichment in the activities of endo-1,4-β-xylanase, α-l-arabinofuranosidase, cellulase, and cellulose binding under the molecular function of the GO category (Fig. 2a, down, red bars with blue dot), suggesting that the transcription of genes that encode cellulolytic enzymes was affected by *Poset2* deletion.

We noticed that the extracellular region was enriched in both glucose and cellulose media (Fig. 2a, red bars with blue star), though the glucose culture was not a good condition for extracellular protein synthesis. Thus, we assayed the extracellular protein expression pattern. Among the 110 secreted proteins determined through *P. oxalicum* secretome analysis [32], 34 secreted protein-encoding genes were differentially expressed (fold change ≥ 2, probability ≥ 0.8) in Δ*Poset2* compared with that in WT when the strains were cultivated in the cellulose medium. We then performed clustering analysis of the differentially expressed genes using the Genesis software. The heatmap showed that 31 (91.2%) of the 34 genes had an upregulated expression (Fig. 2b) and were related to lignocellulose degradation, including 5 genes encoding cellulase (Fig. 2b, pink dots), 24 genes encoding hemicellulase (Fig. 2b, blue dots), and 2 genes encoding expansin (Fig. 2b, yellow triangle).

### Increased production of cellulolytic enzymes was accompanied by the improvement of global H3K4me3 but was not mediated by transcription factors

**Fig. 3:**
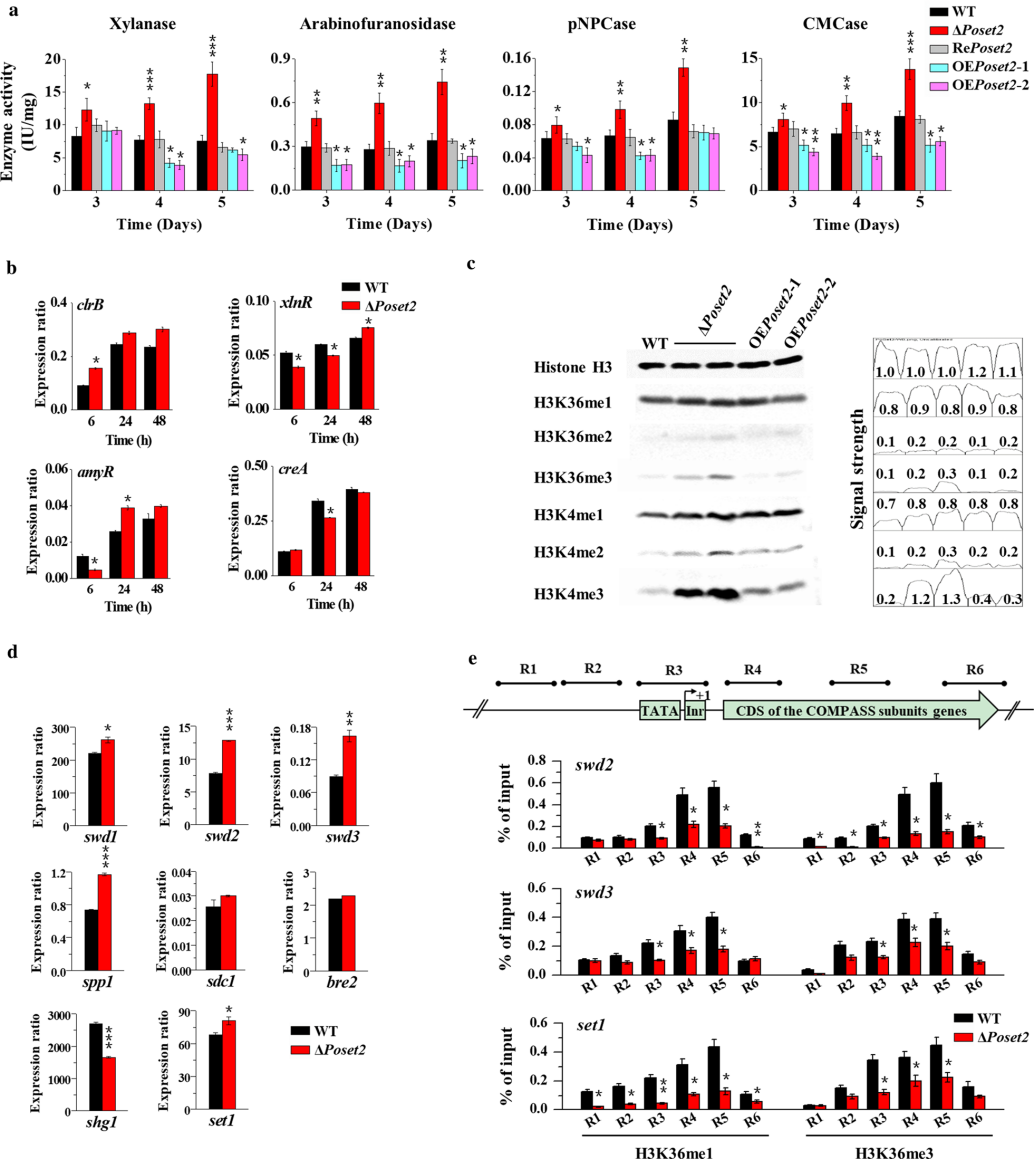
Analysis of enzymatic activity, gene transcription level, and histone methylation patterns in WT and *Po*Set2 mutants. **a** Activity assay of xylanase, arabinofuranosidase, pNPCase, and CMCase. Enzyme activities were normalized to the ratio of corresponding biomass (IU/mg). **b** Expression level assay of genes encoding four transcriptional factors (*clr*-*2*/*clrB*, *xyr1*/*xlnR*, *amyR*, and *cre1*/*creA*) via qRT-PCR analysis. Actin gene was used for data normalization. **c** Analysis of H3K4 and H3K36 methylation patterns through Western blot. Histone H3 was used as the loading control. The peak area and the data were obtained by the software Image J, representing the relative signal strength of the corresponding Western blot bands. **d** Transcript abundance of genes encoding eight subunits (i.e., Swd1, Swd2, Swd3, Bre2, Sdc1, Spp1, Shg1, and Set1) of COMPASS. **e** Analysis of the methylation modification level of H3K36 on the specific regions of three COMPASS subunits encoding genes (*swd2*, *swd3*, and *set1*). The levels of H3K36me1 and H3K36me3 of the target genes were assayed by ChIP-qPCR. Equal amounts of extracted chromatin (1 mg/IP) of each sample were used for IP reactions, and 0.1 mg chromatin DNA was used as input (without IP) for each sample. The purified IP products and input DNA were subjected to quantitative PCR (qPCR). The transcription start site (TSS) was designated as + 1. Six specific regions (R1 to R6) of each gene were designed for qPCR assay (as was shown at the top of the subgraph). Region 1 (R1) and region 2 (R2) were located upstream of the TSS. Region 3 (R3) covered the TATA-box and the initiator (Inr). Region 4 (R4) was located in the 5′ region of the CDS. Region 5 (R5) was located at the middle of the CDS, and region 6 (R6) was located in the 3′ region of the CDS. The relative enrichment of IP DNA was calculated by % of input. The values showed the means of the three biological replicates, and the error bar indicated standard deviation. Statistical significance tests were done by one tailed, unequal variance t-test. *P < 0.05, **P < 0.01, ***P < 0.001

The enzyme activities toward xylan, arabinofuranoside, 4-nitrophenyl β-d-cellobioside (pNPC), and carboxymethylcellulose (CMC) (Fig. 3a) were further analyzed. Enzyme activities were normalized to the ratio of corresponding biomass (IU/mg). The results showed that the activities of these enzymes in Δ*Poset2* increased compared with that in the WT. Specifically, xylanase, arabinofuranosidase, pNPCase (indicating cellobiohydrolase activity), and CMCase (indicating endoglucanase activity) activities of Δ*Poset2* increased by 136.2%, 117.5%, 75.6%, and 66.9% compared with those of WT on day 5, respectively. Re*Poset2* showed almost identical enzyme activities compared with WT. The enzyme activities in *Poset2*-overexpressing strains (OE*Poset2*-1 and OE*Poset2*-2) decreased compared with that in the WT, and the xylanase, arabinofuranosidase, pNPCase, and CMCase activities of OE*Poset2*-2 decreased by 49.7%, 28.5%, 35.2%, and 40.2% compared with those of WT on day 4 (Fig. 3a). The results indicated the increased cellulase and hemicellulase synthesis in Δ*Poset2*.

In fungi, several conserved positive or negative transcriptional factors play key roles in the regulation of cellulolytic enzyme genes, such as positive regulators encoded by *xyr1*/*xlnR* and *clr*-*2*/*clrB* and negative regulators encoded by *cre1*/*creA* and *amyR* [4–6]. We investigated the expression levels of the above key transcription factors (e.g., ClrB, XlnR, CreA, and AmyR) based on quantitative real-time reverse transcription PCR (qRT-PCR) (Fig. 3b). According to our precious investigation about TFs ClrB, CreA, XlnR, and AmyR, the transcriptional-regulatory network of cellulase expression is predicted as a “seesaw model”, in which the coordinated regulation of cellulolytic genes is established by counteracting activators and repressors [6]. Among the selected TFs, two positive regulators encoded by *clrB* and *xlnR* showed different variations (*clrB* slightly upregulated, whereas *xlnR* slightly downregulated). Two negative regulators encoded by *amyR* and *creA* also showed different variations (*amyR* slightly upregulated, whereas *creA* slightly downregulated). Thus, their roles will be interfered with each other. In addition, although the transcription of TFs in Δ*Poset2* and wild-type strains (WT) showed some statistically differences (*P* < 0.05), they were not remarkable enough (*P* > 0.01). Specifically, the transcription of *clrB* did not show any difference (*P* > 0.05) in Δ*Poset2* compared with that in WT at 24 h or 48 h, although ClrB was identified as focal point for the synergistic activation regulation of cellulase expression by integrating cellulolytic regulators [6]. Therefore, the extensive transcriptional activation of cellulolytic enzyme genes caused by the *Poset2* deletion was not mediated by these transcription factors.

Considering that *Po*Set2 was predicted as a typical histone modifier, we determined whether the activation of cellulolytic enzyme expression in Δ*Poset2* was due to the change in histone modification. Therefore, we evaluated monomethylation (H3K36me1), dimethylation (H3K36me2), and trimethylation (H3K36me3) of H3K36 in WT, Δ*Poset2*, and two overexpressing strains (OE*Poset2*-1 and OE*Poset2*-2). We also evaluated monomethylation (H3K4me1), dimethylation (H3K4me2), and trimethylation (H3K4me3) of H3K4 in the WT and the mutants (Fig. 3c). Unexpectedly, Δ*Poset2* displayed almost identical H3K36 methylation patterns compared with that of WT, suggesting that *Po*Set2 was not the sole protein responsible for H3K36 in *P. oxalicum*. Interestingly, H3K4me3 marks in Δ*Poset2* were more abundant (about sixfold) than that of WT as indicated by the obtained signal intensities (Fig. 3c), suggesting that *Poset2* deletion indirectly affected the H3K4 trimethylation level.

In *S. cerevisiae*, H3K4 methylation is usually catalyzed by Set1, which belongs to COMPASS (complex associated with Set1) containing at least eight subunits (i.e., Swd1, Swd2, Swd3, Bre2, Sdc1, Spp1, Sgh1, and Set1) with a remarkable conservation [33]. Most subunits are necessary to efficiently catalyze the methylation of H3K4 [14]. We observed the upregulated expression of most COMPASS subunits (Fig. 3d) in Δ*Poset2*, especially Swd2 and Swd3, upregulated by ~ 64.1% and ~ 83.1%, respectively. Then, three of the eight COMPASS subunits encoding genes (*swd2*, *swd3*, and *set1*), which have significant transcriptional differences in WT and Δ*Poset2*, were selected to conduct detailed methylation analysis (Fig. 3e). For each gene, six typical regions (regions 1 to region 6) distributed upstream and coding domains of the gene were focused. Region 1 (R1) and region 2 (R2) were orderly located at ~ 500 bp upstream (−) of the transcription start site (TSS). Region 3 (R3) covered the initiator and the TATA-box. Region 4 (R4), region 5 (R5), and region 6 (R6) were orderly located in the downstream (+) of TSS, that is, in the 5′ region of the coding domain sequences (CDS), in the middle of the CDS, and in the 3′ region of the CDS (Fig. 3e), respectively. In Δ*Poset2*, decreased levels of H3K36me1 and H3K36me3 were observed in almost all of the detected regions, especially in R3 to R5. The H3K36me3 levels of R5 in *swd2*, *swd3*, and *set1* decreased by 75.1%, 42.9%, and 49.9%, respectively, in Δ*Poset2* compared with that in WT (Fig. 3e). The results were consistent with the absence of H3K36 histone methyltransferase activity due to *Po*Set2 deletion. It suggested that the transcription upregulation of the COMPASS genes in Δ*Poset2* was indeed correlated with the changes in their H3K36 methylation patterns. All in all, *Po*Set2 might negatively regulate the cellulolytic enzyme production by antagonizing the *Po*Set1–H3K4me3 pathway.

### Upregulated expression of cellulolytic enzyme genes was accompanied by the decrease in H3K36 methylation and the increase in H3K4me3 on individual gene loci

**Fig. 4:**
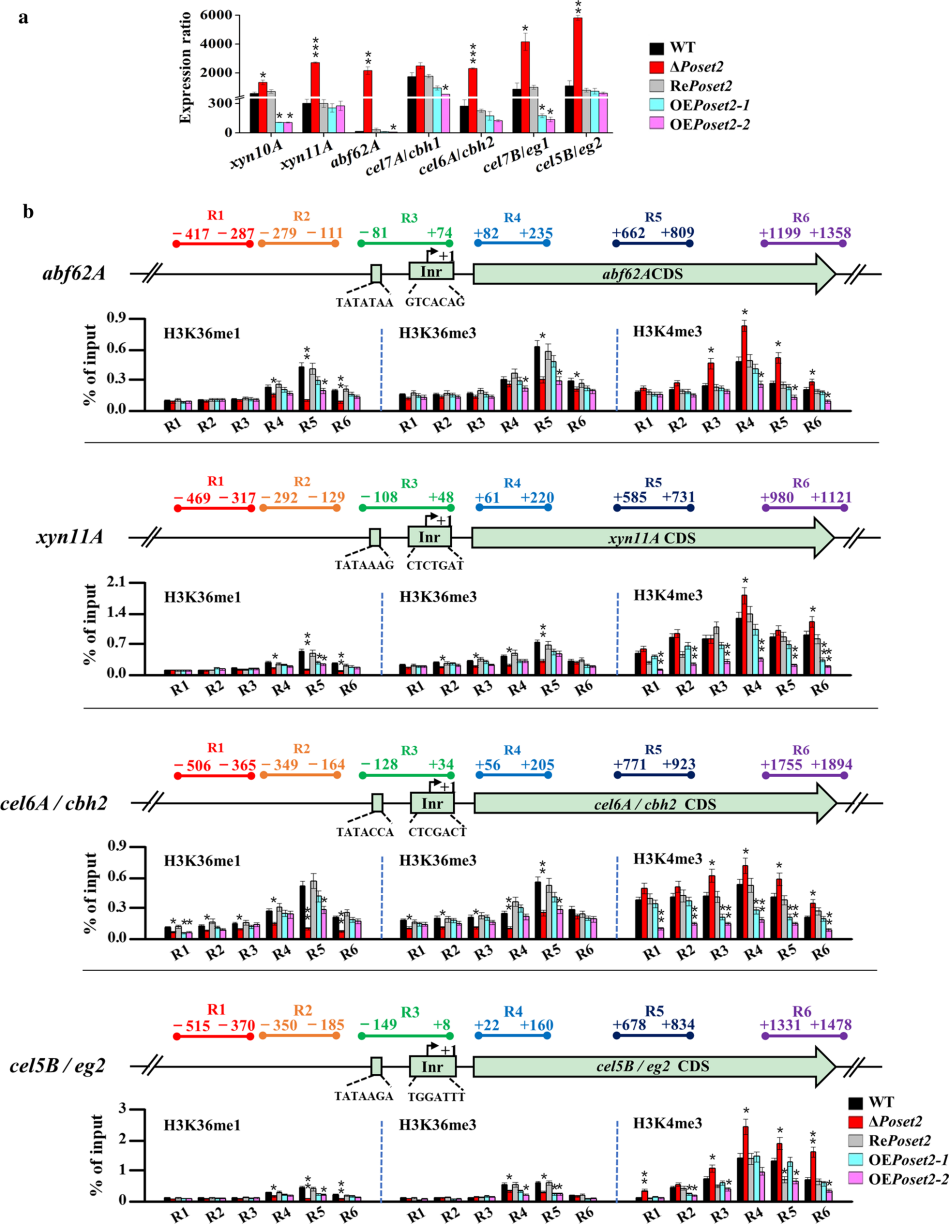
Methylation modification level of H3K4 and H3K36 toward the genes encoding prominent cellulolytic enzymes in WT and *Po*Set2 mutants. **a** The transcript abundance of seven genes encoding prominent cellulolytic enzymes assayed through qRT-PCR. **b** Analysis of the methylation modification level of H3K4 and H3K36 on the specific regions of target genes through ChIP-qPCR. Four genes were selected: two hemicellulase genes, namely, *abf62A* and *xyn11A*, and two cellulase genes, namely, c*el6A/cbh2* and *cel5B/eg2*. The levels of H3K36me1, H3K36me3, and H3K4me3 of the target genes were assayed. The transcription start site (TSS) was designated as + 1. Six specific regions (R1 to R6) of each gene were designed for qPCR assay (top of each subgraph). Region 1 (R1) and region 2 (R2) were located upstream of the TSS. Region 3 (R3) covered the TATA-box and the initiator (Inr). Region 4 (R4) was located in the 5′ region of the CDS. Region 5 (R5) was located at the middle of the CDS, and region 6 (R6) was located in the 3′ region of the CDS. The values showed the means of the three biological replicates, and the error bar indicated standard deviation. Statistical significance tests were performed by one tailed, unequal variance *t*-test. *P < 0.05, **P < 0.01, ***P < 0.001

The results of Western blot analysis showed the global levels of histone methylation that could not represent the chromatin modification at specific gene loci. Thus, the local analysis of H3K36 and H3K4 methylation at specific cellulolytic gene loci was necessary. We detected the transcripts of major hemicellulase-encoding genes (i.e., *xyn10A*, *xyn11A*, and *abf62A*) and the cellulase encoding genes (i.e., *cel7A*/*cbh1*, *cel6A*/*cbh2*, *cel7B*/*eg1*, and *cel5B*/*eg2*) via qRT-PCR analyses. The high transcript abundance of these genes was observed in Δ*Poset2* compared with that in WT (Fig. 4a). Then, four genes with significantly upregulated transcription levels, namely, hemicellulase genes *abf62A* (~ 200 times) and *xyn11A* (~ 8.3 times), and cellulase genes *cel6A*/*cbh2* (~ 6.1 times) and *cel5B*/*eg2* (~ 5.5 times), were selected as the targets for histone methylation level analysis at the individual gene loci. For each gene, six typical regions (Region 1–Region 6) as were designed in the ChIP-qPCR for COMPASS genes, were also designed and focused in the ChIP-qPCR for cellulolytic enzyme encoding genes.

In WT, the low levels of H3K36 methylation were detected in region 1, region 2, and region 3, whereas the highest methylation level was detected in region 5. The result was expected because H3K36 methylation is associated with coding regions and transcription elongation [34]. In Δ*Poset2*, the decreased levels of H3K36me1 and H3K36me3 were observed in all of the detected regions, especially in region 5. The H3K36me1 levels of region 5 in *abf62A*, *xyn11A*, *cbh2*, and *eg2* decreased by 76.4%, 77.1%, 80.2%, and 80.1%, respectively, in Δ*Poset2* compared with that in WT (Fig. 4b). In contrast to the significant decrease in H3K36 methylation, the levels of H3K4me3 were improved in all of the selected gene regions in Δ*Poset2*, especially in region 4. The H3K4me3 levels in the region 4 of *abf62A*, *xyn11A*, *cbh2*, and *eg2* improved by 72.5%, 40.3%, 33.6%, and 70.8%, respectively, in Δ*Poset2* compared with that in WT (Fig. 4b). In addition, significant decreases of H3K4me3 on specific cellulolytic gene loci were observed in OE*Poset2* (Fig. 4b). Considering that H3K4me3 was an active marker for the expression of cellulolytic enzyme encoding genes, the decreased H3K4me3 on specific cellulolytic gene loci could result in decreased cellulolytic enzyme expression in OE*Poset2*. These findings suggested that the trimethylation of H3K4 was mainly enriched during the transcription initiation in the 5′ region of the CDS (region 4), and *Po*Set2 negatively affected the trimethylation of H3K4 in the selected targets.

### H3K4 methylation performed by *Po*Set1 was essential for cellulolytic enzyme production

**Fig. 5:**
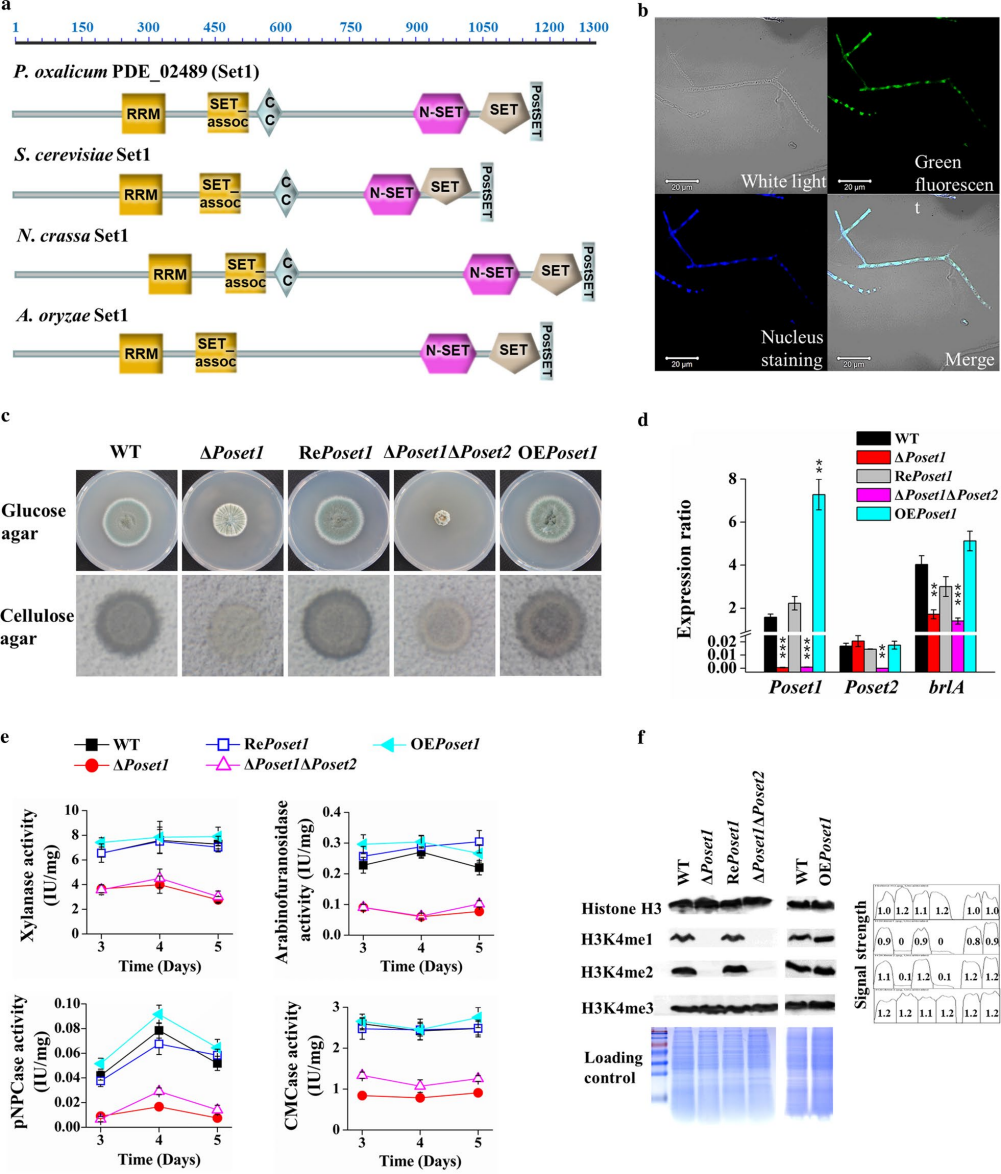
Phenotypic analysis of *Po*Set1 mutants. **a** Domain architecture analysis of *Po*Set1 orthologs. **b** Subcellular localization of *Po*Set1 in *P. oxalicum*. Upper left, white light; upper right, green fluorescence (green dots); bottom left, nuclear staining (blue dots); bottom right, merged (white dots). **c** Morphological characteristics of the colony of 4-day-old cultures on Vogel’s agar with 2% glucose or 0.5% ball-milled microcrystalline cellulose. **d** Assay of the transcription level of *brlA* through RT-qPCR. **e** Activity assay of xylanase, arabinofuranosidase, pNPCase, and CMCase in WT and *Po*Set1 mutants. Enzyme activities were normalized to the ratio of corresponding biomass (IU/mg). Statistical significance tests were performed by one tailed, unequal variance *t*-test. *P < 0.05, **P < 0.01, ***P < 0.001. **f** Analysis of H3K4 methylation patterns through Western blot. Histone H3 was used as the loading control. The peak area and the data were obtained by the software Image J, representing the relative signal strength of the corresponding Western blot bands

The increased H3K4me3 and the upregulated expression of most COMPASS subunits were observed in Δ*Poset2* (Fig. 3c, d). To understand whether this phenomenon was associated with the upregulation of cellulolytic enzymes, we investigated *Po*Set1, which is responsible for H3K4 methylation in COMPASS. *Po*Set1 contains typical domains that are presented in other fungal Set1 homologs (Fig. 5a) and located in the nucleus (Fig. 5b). In comparison with WT, the lack of *Po*Set1 (Δ*Poset1*) resulted in a smaller colony diameter, few spores (Fig. 5c), and declined transcription of sporogenesis-related *brlA* (Fig. 5d). The *Poset1*-overexpressing strain (OE*Poset1*) had a phenotype identical to that of WT (Fig. 5c, d).

When the mutants were cultivated in cellulose agar, no cellulolytic halo was found around the Δ*Poset1* colony (Fig. 5c). The cellulolytic halo also disappeared in Δ*Poset1*Δ*Poset2* (Fig. 5c), although the single-deletion mutant Δ*Poset2* could cause a more highly pronounced halo than that of WT as shown in our previous works (Fig. 1c). The activities of xylanase, arabinofuranosidase, pNPCase, and CMCase in Δ*Poset1* decreased remarkably to 38.0%, 22.2%, 32.4%, and 14.5% of that in WT. Almost the same degree of decline was shown in the double-deletion mutant Δ*Poset1*Δ*Poset2* (Fig. 5e), although the enzyme activities in Δ*Poset2* increased compared with those in WT (Fig. 3a). We did not observe any significant change in terms of enzyme activities in *Poset1*-overexpressing strains (Fig. 5c, e). The results suggested the important role of *Po*Set1 in cellulolytic enzyme production. The upregulation of cellulolytic enzymes caused by *Po*Set2 deletion relied on the existence of *Po*Set1.

The repressed cellulolytic enzyme production observed in Δ*Poset1* and Δ*Poset1*Δ*Poset2* (Fig. 5e) was accompanied by the absence of H3K4me1 and H3K4me2 signals (Fig. 5f), suggesting that *Po*Set1 is the sole methyltransferase responsible for H3K4 monomethylation and dimethylation. The block of H3K4me2 was also observed in *Magnaporthe oryzae* Set1 deletion strain with the repressed cellulase gene *cel7C* [35]. The increased signal strength of H3K4me3 previously observed in Δ*Poset2* (Fig. 3c) was repressed when *Po*Set1 was absent in Δ*Poset2* background (Δ*Poset1*Δ*Poset2*) (Fig. 5f). This suggested that the increased H3K4me3 signal strength caused by *Poset2* deletion relied on the function of *Po*Set1. In addition, the OE*Poset1* had identical H3K4 methylation signal strength to that of WT (Fig. 5f).

### Decrease of H3K4me3 in specific gene sites accompanying the downregulated expression of cellulolytic enzyme genes

**Fig. 6:**
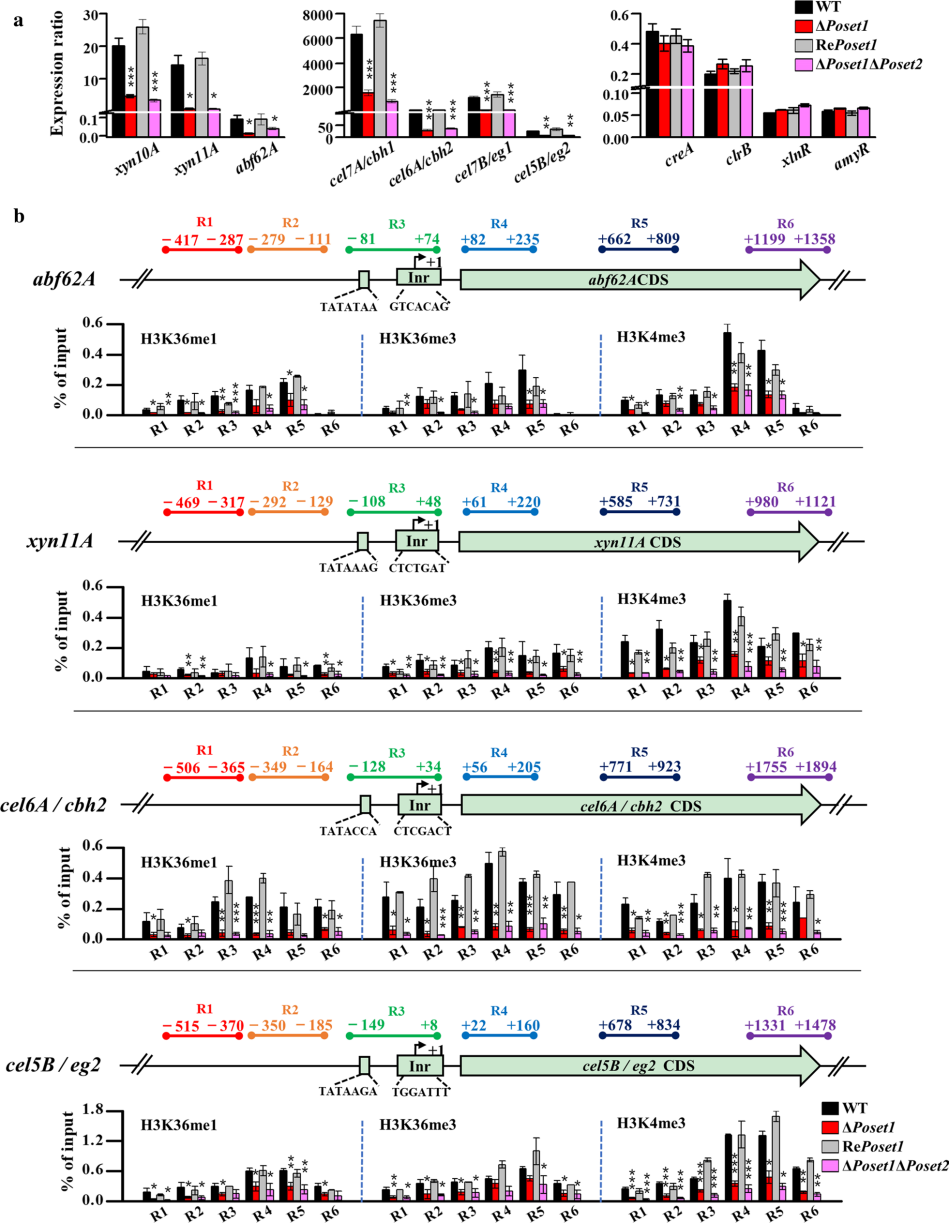
Methylation modification level of H3K4 and H3K36 toward genes encoding prominent cellulolytic enzymes in WT and *Po*Set1 mutants. **a** The transcript abundance of seven genes encoding prominent cellulolytic enzymes and four genes encoding TFs were assayed through qRT-PCR. The diagram on the left, three genes (*xyn10A*, *xyn11A*, and *abf62A*) encoding hemicellulases; the diagram in the middle, four genes (*cel7A*/*cbh1*, *cel6A*/*cbh2*, *cel7B*/*eg1*, and *cel5B*/*eg2*) encoding cellulases; the diagram on the right, four genes (*creA*, *clrB*, *xlnR*, and *amyR*) encoding TFs. **b** Analysis of the methylation modification level of H3K4 and H3K36 on the specific regions of target genes through ChIP-qPCR. Four genes were selected: two hemicellulase genes, namely, *abf62A* and *xyn11A*, and two cellulase genes, namely, c*el6A/cbh2* and *cel5B/eg2*. The levels of H3K36me1, H3K36me3, and H3K4me3 of the target genes were assayed. The design of the six regions (R1–R6) of each gene locus is the same as the figure legend described in Fig. 4b. Relative enrichment of IP DNA was calculated as the percentage of the total input DNA. The values show the mean of the three biological replicates, and the error bar indicates standard deviation. Statistical significance tests were performed by one tailed, unequal variance *t*-test. *P < 0.05, **P < 0.01, ***P < 0.001

We found that the transcription of seven major cellulolytic enzyme genes showed a remarkable decrease in both Δ*Poset1* and Δ*Poset1*Δ*Poset2* (Fig. 6a). For example, the transcripts of *abf62A*, *xyn11A*, *cbh2*, and *eg2* in Δ*Poset1* decreased to only 14.1%, 5.5%, 16.4%, and 24.1% compared with that in the WT (Fig. 6a). The expression level of the TFs (*xlnR*, *clrB*, *creA*, and *amyR*) was also tested in *Poset1*-associated mutants by RT-qPCR. Expression levels of all the four TFs did not show significant statistical difference (*P* > 0.05) according to the results of statistical analysis (Fig. 6a). It suggested that the decrease in expression in target enzymes was not due to changes of the TFs. The levels of the three kinds of histone H3 methylation (i.e., H3K36me1, H3K36me3, and H3K4me3) at specific gene loci, which we examined in Δ*Poset2* (Fig. 4b), were also detected in Δ*Poset1* and Δ*Poset1*Δ*Poset2* (Fig. 6b). The decrease in H3K4me3 at the individual gene loci was observed in Δ*Poset1*, which was especially significant in region 4 corresponding to the 5′ region of the CDS. In particular, the H3K4me3 levels in region 4 of *abf62A*, *xyn11A*, *cbh2*, and *eg2* in Δ*Poset1* decreased to only 33.9%, 30.8%, 15.8%, and 26.5% of that in WT, respectively (Fig. 6b). In contrast to *Po*Set2 antagonizing trimethylation of H3K4, *Po*Set1 was assumed as an indirect agonist of H3K36 methylation because the decrease in H3K36me1 and H3K36me3 at the individual gene loci was also observed in Δ*Poset1*.

Both H3K36me1 and H3K36me3 decreased at the individual gene loci in Δ*Poset1*Δ*Poset2*. This result is consistent with that of Δ*Poset2*. However, the upregulation effect on H3K4me3 at the individual gene loci caused by *Poset2* deletion was eliminated when *Po*Set1 was absent on the Δ*Poset2* background. In particular, the H3K4me3 levels in the region 4 of *abf62A*, *xyn11A*, *cbh2*, and *eg2* in Δ*Poset1*Δ*Poset2* decreased to 29.9%, 15.1%, 17.8%, and 18.2% of that in WT, respectively (Fig. 6b), accompanying the downregulated expression of the cellulolytic enzyme genes (Fig. 6a). This finding implied that H3K4me3 enrichment was an active marker of the cellulolytic enzyme gene. The upregulation of cellulolytic enzymes in Δ*Poset2* relied on the increased H3K4me3 performed by *Po*Set1. As a whole, the results showed that the balance of H3K4 and H3K36 methylation induced by *Po*Set1 and *Po*Set2 is required for the normal transcription of cellulolytic enzyme genes.

## Discussion

**Fig. 7:**
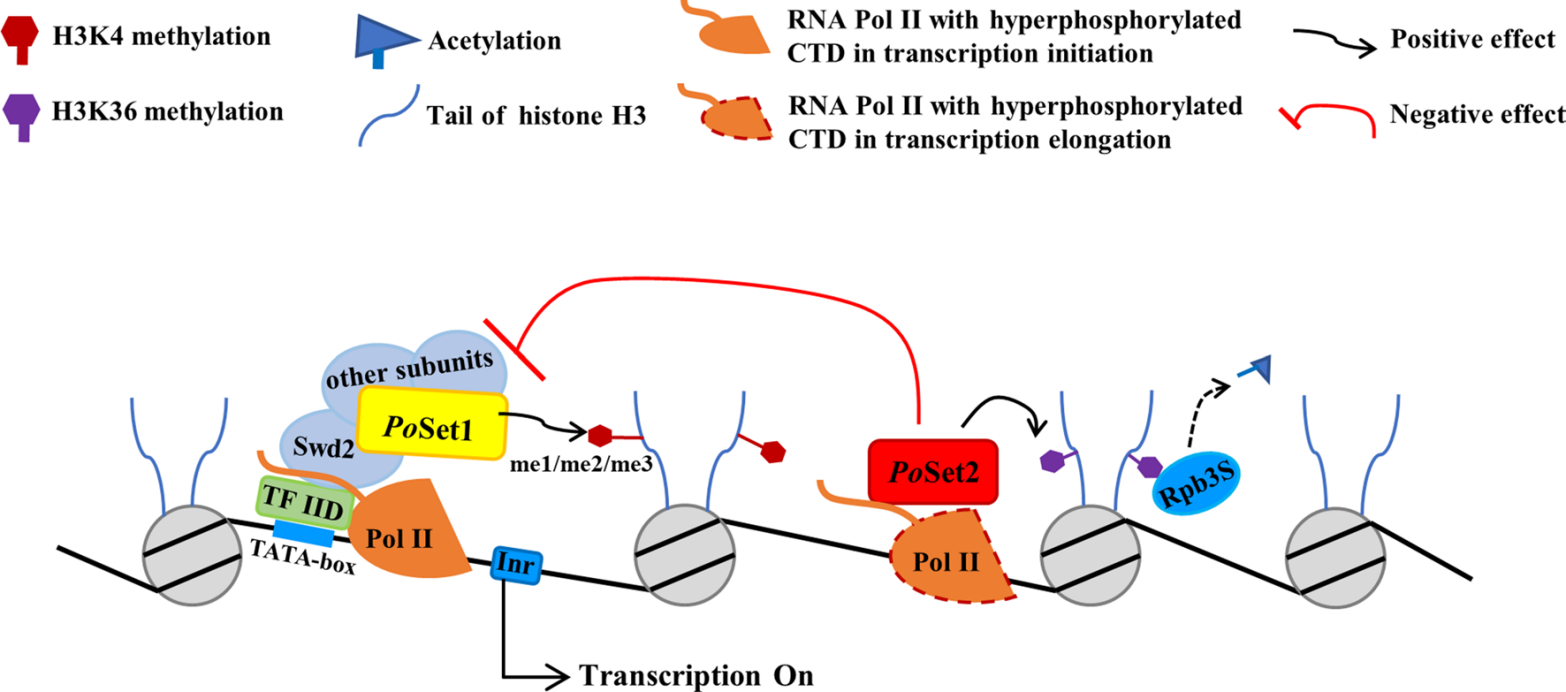
Putative regulatory pattern of *Po*Set1 and *Po*Set2 on cellulolytic enzyme gene expression. *Po*Set1 is the sole histone methyltransferase responsible for H3K4me1 and H3K4me2 in *P. oxalicum*. *Po*Set1 was preferentially located in the core promoter of target genes via the interactions with other COMPASS subunits (i.e., Swd1, Swd2, Swd3, Bre2, Sdc1, Spp1, and Sgh1), Pol II and general transcription factors, such as TFIID. The methylation of H3K4 is an active marker for the transcription initiation of cellulolytic enzyme genes. *Po*Set2 is a H3K36 methyltransferase that coordinates transcriptional elongation at the coding regions. The methylation of H3K36 is a repression marker of the transcription of cellulolytic enzyme genes. The repressive Rpd3S complex was recruited by methylated H3K36 and promoting histone tail deacetylation [38]. *Po*Set2 antagonized the *Po*Set1–H3K4me3 pathway by negatively regulating the expression of COMPASS subunits. The balance of H3K36 and H3K4 methylation is required for the normal transcription of cellulolytic enzyme genes

Our results demonstrated that the activated cellulolytic enzyme genes were accompanied by increase of H3K4me3 and decrease of H3K36me1 and H3K36me3 on specific gene loci. Correspondingly, the repression of cellulolytic enzyme genes was accompanied by the absence of the global H3K4me1 and H3K4me2 and the decrease of H3K4me3 on specific gene loci. Furthermore, the increase in the H3K4me3 level in Δ*Poset2* was eliminated in Δ*Poset1*Δ*Poset2* accompanied by the repressed cellulolytic enzyme genes. Therefore, we proposed a model (Fig. 7) in which H3K4 methylation was an active marker of cellulolytic enzyme production, whereas H3K36 methylation was a marker of repression. A crosstalk occurred between H3K36 and H3K4 methylation, and *Po*Set2 antagonized the *Po*Set1–H3K4me3 pathway. The balance of H3K4 and H3K36 methylation was required for the normal transcription of cellulolytic enzyme genes.

Our initial speculation indicated that *Po*Set2 might be a positive regulator of cellulolytic enzyme gene transcription because the methylation of H3K36 is commonly associated with transcriptional activation [36, 37]. Therefore, it was unexpected that the active cellulolytic enzyme genes of WT were accompanied by the decreased H3K36 methylation, and the decrease in H3K36 methylation on specific cellulolytic enzyme gene loci in Δ*Poset2* was accompanied by the upregulated transcription of cellulolytic enzyme genes. Studies have demonstrated that Set2 and H3K36 methylation has also been implicated in transcriptional repression; for example, Set2 inhibits transcriptional initiation by recruiting a repressive Rpd3S complex and promoting histone tail deacetylation [38, 39]. In addition, the transcriptome results (Δ*Poset2* vs. the WT) indicated the downregulated (~ 43.5%) expression of *hepA* (data not shown), whose ortholog Tup1 in budding yeast resulted in the Rpd3S binding to H3K36me2/3 through its plant homeobox domain (PHD) finger [40]. Therefore, the impaired methylation of H3K36 on specific cellulolytic enzyme gene loci and the corresponding repression effect on the Rpd3S complex might be one of the reasons for the activated cellulolytic enzyme gene in Δ*Poset2*. However, we could not assume that this result was the most important reason for the activation of cellulolytic enzyme gene because H3K36 methylation on the specific gene loci in Δ*Poset1*Δ*Poset2* also decreased, but this decrease was accompanied by the repressed expression of cellulolytic enzyme genes.

The results suggested that H3K4 methylation was required for the transcriptional activation of cellulolytic enzyme genes. Set1, responsible for H3K4 methylation, is the core subunit of COMPASS. When we identified the protein–protein interaction collaborator of *Po*Swd2, another subunit of COMPASS, using tandem affinity purification/mass spectrometry (TAP-MS) experiments, most subunits of COMPASS (i.e., Swd1, Swd2, Swd3, Bre2, Sdc1, Spp1, and Set1), subunits of Pol II (i.e., Pol II subunit A, subunit B, subunit C, and subunit E), and general transcription factor TFIID, were captured (Additional file 6: Figure S3; Additional file 7: Spreadsheet S3). As TFIID was a critical factor in promoter recognition and preinitiation complex establishment [41], and the enrichment of H3K4 methylation was observed in region 3 (core promoter) and region 4 (5′-CDS) on specific cellulolytic gene loci, *Po*Set1 likely participated in the transcription initiation of cellulolytic enzyme gene, whose process was mediated by the interactions among *Po*Set1, other COMPASS subunits, and general transcription factor.

Full genome scan of histone methylation in yeast has revealed that H3K4me2 and H3K4me3 predominantly accumulate in transcriptionally active genes [42]. Specifically, H3K4me3 is a mark found at open and potentially active promoters [43]. Our results also supported that there is an enrichment of H3K4me3 in the regions (region 3 and region 4) covering the TATA-box and the initiator of active gene (Fig. 4b), as well as a decrease of H3K4me3 in repressed gene (Fig. 6b). We also noticed a global depletion of H3K4me1 and H3K4m2 methylation in Δ*Poset1* (Fig. 5f). H3K4me2 is often detected in the coding regions [44]. Carboxymethylcellulose-induced *M. oryzae* cellulose *cel7C* gene activation was drastically diminished in a knockout mutant of the MoSET1 gene, accompanying with an undetectable level of H3K4me2 [35]. Thus, the repressed cellulolytic enzyme genes in Δ*Poset1* might be affected in the process of transcriptional initiation and elongation simultaneously. In addition, it was reported that H3K4 methylation shows specific enrichment patterns among genes. There exists apparent discrepancy of gene expression and significant changes in H3K4-me2 or -me3 abundance during infection-related morphogenesis in *M. oryzae* [24]. Therefore, a global scale of RNA-seq and ChIP-seq analysis should be done in future to examine whether changes in the H3K4me1/me2/me3 patterns were associated with gene activation or silencing in Δ*Poset1*.

Considering that H3K4me3 was enriched by *Po*Set2 deletion, whereas the enriched H3K4me3 was eliminated by further *Po*Set1 deletion in Δ*Poset2*Δ*Poset1*, we hypothesized that *Po*Set2 antagonized the *Po*Set1–H3K4me3 pathway. Indeed, we observed the upregulated expression of most COMPASS subunits in Δ*Poset2*, especially Swd2 and Swd3 (Fig. 3d). Swd2 is a major H3-binding COMPASS component representing a key factor that mediates a crosstalk between H2B ubiquitylation and H3K4 trimethylation on chromatin [45]. Swd3 is essential for the integrity of COMPASS and the stability of Set1 [46]. *Po*Set2 may antagonize the *Po*Set1–H3K4me3 pathway by negatively affecting the expression of these COMPASS subunits. In addition, when *Po*Set1 is overexpressed alone, we did not observe the significant upregulation of H3K4 methylation and cellulolytic gene expression. This finding implied that *Po*Set1 must interact with other subunits as a complex to perform methylation. The co-upregulation of these subunits in Δ*Poset2* promoted the function of COMPASS, further resulting in the increased H3K4me3.

Although H3K36 methylation obviously decreased in specific cellulolytic enzyme gene loci, the global change in H3K36 methylation was not observed in Δ*Poset2*. Indeed, the global and gene-specific histone modification patterns are inconsistent in some instances [47]. In contrast to the sole methyltransferase Set2 responsible for H3K36 methylation in budding yeast, three methylation events at H3K36 require a division of methyltransferases between monomethylases and dimethylases and the SET2-type trimethylases in higher eukaryotes [26, 48]. For example, two Set2 homologs, namely, Sdg8 and Sdg26, in *Arabidopsis thaliana* regulate distinct degrees of H3K36 methylation [49]. *Po*Set1 is the sole enzyme responsible for H3K4me1 and H3K4me2 in *P. oxalicum*, while other methyltransferases that perform H3K4 trimethylation may exist. Upon reexamining the SET domain-containing proteins, we noticed that PDE_01581 contained SET and PS domains but not AWS or WW domains. PDE_01581 was originally disregarded because it has no SRI domain, which is responsible for the interaction between Set2 and RNA Pol II and couples histone H3 K36 methylation with transcript elongation [50]. The high degree of homology between PDE_01581 and Ash1 was observed. The mammalian Ash1L is responsible for the monomethylated and dimethylated H3K36 [51] and the trimethylated H3K4 [52]. Therefore, the protein PDE_01581 might be a histone methyltransferase that worked with H3K4 and H3K36 as substrates. Nevertheless, this finding should be investigated in the future.

## Conclusions

In summary, the results obtained in this study indicate that H3K4 methylation was an active marker of cellulolytic enzyme production, whereas H3K36 methylation was a marker of repression. A crosstalk occurred between H3K36 and H3K4 methylation, and *Po*Set2 negatively regulates cellulolytic enzyme production by antagonizing the *Po*Set1–H3K4me3 pathway. The balance of H3K4 and H3K36 methylation was required for the normal transcription of cellulolytic enzyme genes. Active or repressed genes are not always directly associated with TFs. These results extend our previous understanding that cellulolytic enzyme gene transcription is primarily controlled by TFs.

## Methods

### Fungal strains and culture conditions

Wild-type (WT) *P. oxalicum* 114-2 (CGMCC 5302) was used as the original parent strain. In accordance with the principle of homologous recombination, the single-deletion mutants Δ*Poset2* and Δ*Poset1* were constructed on the basis of WT, and the double-deletion mutant Δ*Poset1*Δ*Poset2* was constructed on the basis of Δ*Poset2*. In accordance with the principle of random insertion, the re-complement strains were constructed with the corresponding fontal promoters based on the deletion mutants, and the overexpressed strains were constructed with the strong promoter *gpdA* based on WT. The mutants were further verified using diagnostic PCR and Southern blot analysis. According to the principle of complementary hybridization, particular stripes in the nylon membrane (PALL, Ann arbor, USA) were visualized using a DIG DNA labeling system (Roche, Indianapolis, USA). The probes were labeled via PCR using a PCR DIG Probe Synthesis kit (Roche, Indianapolis, USA). The strategies and results of PCR and Southern blot are presented in Additional file 8: Figure S4. The primers used here are shown in Additional file 9: Table S2.

The strains were grown in either 1× Vogel’s agar with 2% glucose (w/v), 1% starch, or 0.5% ball-milled cellulose as a sole carbon source or a fermentation medium with 2% glucose (glucose medium) or 1% microcrystalline cellulose (Sangon, Shanghai, China, CB0279) plus 1% wheat bran (cellulose medium) as carbon sources as previously described [53] and cultivated at 30 °C for 3–5 days. Three replicates were included for each sample.

### Phylogenetic tree and domain architecture analyses

Amino acid sequences of Set2 and Set1 from different fungal species were obtained from NCBI [54]. For the phylogenetic tree analysis, multiple sequences alignment was performed using software Clustal X [55] and MEGA7.0 [56], respectively. For domain architecture analysis, we referred to the websites SMART [57, 58] and Pfam database [59, 60]. Structure domain comparison maps were constructed with equal proportion of the respective sequences.

### Phenotype analysis and microscopic observation

To observe colony morphology and conidiation of different mutants, fresh spore solutions with equal spore concentrations (10^7^/mL) were prepared. 1.5 μL of each solution were spotted on Vogel’s medium ager plates with 2% glucose, 1% starch, or 0.5% ball-milled cellulose as sole carbon source, and then cultivated at 30 °C for 3–5 days. Hydrolysis circles in 1% starch or 0.5% ball-milled cellulose were observed by adding 0.5% Triton X-100 to limit the elongation of hypha. Iodine solution containing 6 g KI and 0.6 g I_2_ in 100 mL of H_2_O was added to the surface of 1% starch medium at room temperature for 10 min to visualize starch-degrading circles.

To count the spore yield of different mutants, 100 μL of spore solutions were evenly coated to Vogel’s medium plates with 2% glucose as carbon source, and were then incubated at 30 °C for 5 days. Ten-mm-diameter colony agar plugs were sampled for each strain in triplicates. The numbers of conidia were counted using a hemocytometer. The method of cover slip culture and aniline blue staining was used for conidiophores observation [31]. Briefly, several sterile cover slips were placed into the medium at a 45 angle to the agar surface and incubated at 30 °C. Then, cover slips were removed at the designated time points and were stained with lactophenol cotton blue reagent (0.05-g cotton blue, 20.0-g phenol crystals, 40.0-mL glycerol, 20.0-mL lactic acid, and 20.0-mL distilled water). The nature of conidiophores and conidial shapes were observed using Nikon phase-contrast microscope. To observe intracellular localization of *Po*Set2 and *Po*Set1, the mycelia on the cover slip were stained with 1 µg/mL Hoechst 33342 for 15 min in the dark to obtain nucleus staining. The slips were viewed under high-sensitivity laser scanning confocal microscope (ZEISS LSM780) (Carl Zeiss, Oberkochen, Germany). The excitation light at 488 nm wavelength was used for the green fluorescence of *Po*Set2–GFP or *Po*Set1–GFP, and at 405 nm for nuclear staining.

### Cellulase and hemicellulase activity assay

The culture supernatants (crude enzyme) from different time points were collected via centrifugation and diluted with 0.2-M NaAc buffer solution (pH 4.8). The activities of endoglucanase (CMCase) and xylanase were assayed in accordance with the methods described by Li et al. [6]. Carboxymethylcellulose sodium salt (CMC-Na) (Sigma-aldrich, St. Louis, USA) and xylan (Ryon, Shanghai, China) were used as substrates. Briefly, 0.5 mL of suitably diluted culture supernatants and 1.5 mL of 1% (m/v %) CMC-Na or 1% (m/v %) xylan, which was dissolved in NaAc buffer (pH 4.8), were added into 25-mL colorimetric tubes. The mixture was gently stirred and incubated in a 50 °C water bath for 30 min. 3 mL 3,5-dinitrosalicylic acid (DNS) reagent (10 g 3, 5-dinitrosalicylic acid, 20-g sodium hydroxide, 200-g sodium potassium tartrate, 2.0-g redistilled phenol, and 0.50-g sodium sulfite anhydrous per 1000-mL DNS reagent) were then added to stop enzymatic reaction. The tubes were placed in boiling water for 10 min. Finally, 20 mL of distilled water was added and mixed, and the absorbance of the reaction mixture was measured at 540 nm to determine enzyme activities. The cellobiohydrolase (pNPCase) and α-l-arabinofuranosidase activities were measured using 1 mg/mL pNPC (Sigma-aldrich, St. Louis, USA) and 1 mg/mL 4-nitrophenyl α-l-arabinofuranoside (Carbosynth, Oxford, UK) as substrates, respectively. Briefly, 100 μL of suitably diluted culture supernatants and 50 μL of substrates were gently mixed and incubated at 50 °C for 30 min. 150 μL of 10% Na_2_CO_3_ (w/v) was then added to terminate the reaction, and the absorbance of the reaction mixture was measured at 420 nm to determine the enzyme activities. One enzyme activity unit was defined as the amount of enzyme required for producing 1 μmol glucose or pNP per minute under the assayed conditions.

Cellulolytic enzyme activities were normalized to the ratio of corresponding biomass. Here, it was not appropriate to assay the mycelia biomass according to the dry cell weight, because cellulose and wheat bran were added in the fermentation medium as inducer and unconsumed cellulose and wheat bran interfered with the results. Thus, concentration of total intracellular protein was determined to indicate the biomass using the methods previously described by Zhang et al. [61]. The normalized cellulolytic enzyme activities (IU/mg) represented the enzyme activity produced by the mycelium corresponding to 1-mg intracellular protein. Three biological triplicates were performed for all enzyme analysis. The mean values and standard deviations were calculated.

### Quantitative real-time reverse transcription PCR (qRT-PCR) analysis

Total RNA was extracted from mycelia after 6, 24, or 48 h of cultivation in a cellulose medium with RNAiso Plus reagent (TaKaRa, Kusatsu, Japan). Genomic DNA was removed and cDNA was synthesized using a PrimeScript™ RT reagent kit (TaKaRa, Kusatsu, Japan). The obtained cDNA was used as a template of quantitative PCR through LightCycler^®^ 480 (Roche, Indianapolis, USA) with SYBR Premix Ex Taq™ (TaKaRa, Kusatsu, Japan). The copy number of unambiguous transcripts for each gene was normalized to expression ratio by the transcripts of *actin*. The primers used were shown in Additional file 9: Table S2.

### RNA sequencing and GO analysis

Mycelia of WT and mutants were collected after 24 h of fermentation cultivation. Total RNA was extracted with methods described in “Quantitative real-time reverse transcription PCR (qRT-PCR) analysis” section. The extracted RNA was incubated with 10 U DNase I (Takara, Dalian, China) at 37 °C for 30 min to remove genomic DNA. RNA was assayed to ensure its quality (OD260/OD280: 1.8–2.2; OD260/OD230: > 1.5; RNA Integrity Number (RIN): > 8.0) before subsequent library construction. Transcriptome assay based on BGISEQ-500 RNA-Seq was performed by the Beijing Genomics Institute (BGI, Shenzhen, China). The dataset was generated by paired-end (PE) modes. Sequenced reads were mapped against predicted transcripts and reference genome using Bowtie2 [62] and HISAT [63], respectively. Then, the filtered clean reads for each gene were normalized to fragments per kilobase transcriptome per million mapped reads (FPKM) by using Cufflinks [64]. Gene expression level was quantified by RSEM software package [65]. Significantly different expression levels between samples were identified through a significance test with combined thresholds (diverge probability ≥ 0.8 and fold change ≥ 2), in which the probability value represented the probability of the significance of different genes (the larger this value was, the more significant it was) [66]. Cluster analysis and function enrichment analysis were conducted in Genesis [67] and Blast2GO with a threshold at FDR ≤ 0.05 [68].

### Western blot analysis

Total protein was extracted from the mycelia after 24 h of fermentation cultivation by mixing with the extraction buffer containing 50-mM Tris–HCl (pH 7.5), 150-mM NaCl, 1% NP-40, and 1-mM PMSF in an ice bath for 30 min and via centrifugation at 12,000 rpm at 4 °C for 10 min to collect the supernatant with the total protein. Equal amounts of total protein were separated by SDS polyacrylamide gel electrophoresis (SDS-PAGE) on a 15% gel and were transferred to nitrocellulose membrane (PALL, Ann arbor, USA) using a Bio-Rad electroblotting apparatus. Anti-H3K4me1 antibody (ab8895, Abcam, Cambridge, UK), anti-H3K4me2 antibody (A2356, ABclonal, Wuhan, China), and anti-H3K4me3 antibody (ab8580, Abcam, Cambridge, UK) were utilized to detect H3K4 methylation. Anti-H3K36me1 antibody (OM256826, OmnimAbs, California, USA), anti-H3K36me2 antibody (A2365, ABclonal, Wuhan, China), and anti-H3K36me3 antibody (ab9050, Abcam, Cambridge, UK) were employed to detect H3K36 methylation. Equal amounts of the total protein and the anti-histone H3 antibody (OM256785, OmnimAbs, California, USA) were set as the loading control. The signal strength of Western blot band was quantified by the software Image J.

### Chromatin immunoprecipitation-quantitative PCR (ChIP-qPCR)

ChIP assays were performed in accordance with a previously described protocol [69]. Briefly, the hyphae cultivated in liquid fermentation medium were fixed by the adding of 1% formaldehyde at 30 °C for 10 min with shaking, followed with 125-mM glycine shaking for an additional 5 min. All hyphae were collected and grounded to powder in liquid nitrogen, and fully lysed in lysis buffer (50-mM HEPES pH 7.5, 150-mM NaCl, 1-mM EDTA, 0.5% Triton X-100, 0.1% sodium deoxycholate, 0.1% SDS, 1-mM PMSF (phenylmethanesulfonyl fluoride), 0.1% protease inhibitor cocktail). The chromatin was obtained by centrifugation and was further broken into fragments of approximately 500 bp by sonication. Immunoprecipitation (IP) was performed with anti-H3K36me1 antibody (OM256826, OmnimAbs, California, USA), anti-H3K36me3 antibody (OM256832, OmnimAbs, California, USA), and anti-H3K4me3 antibody (OM256846, OmnimAbs, California, USA) with equal amounts of extracted chromatin (1 mg). The obtained IP products and 0.1-mg input chromatin DNA (without IP) of each sample were subjected to RNase digestion to remove RNA, as well as high heat and proteinase K digestion to reverse crosslinks, followed by phenol–chloroform extraction and ethanol precipitation to get purified IP DNA and input DNA. Quantitative PCR was finally performed by using LightCycler^®^ 480 (Roche, Indianapolis, USA). The relative enrichment of IP DNA was defined as the ChIP efficiency and calculated as a percentage of the total input DNA by using the following equation, where Ct = threshold cycle of PCR. All of the primers used here are shown in Additional file 9: Table S2.

$${{\text{ChIP}}}\;{{\text{efficiency}}} = 2^{{{ - \Delta {\text{Ct}}}}} \times 100\%$$

$$\Delta {\text{Ct}} = {\text{Ct}}_{\text{IP}} - \left( {{\text{Ct}}_{\text{Input}} - \log_{2} 10} \right)$$

### Statistical significance tests

All statistical significance tests were done with a one-tailed homoscedastic (equal variance) *t*-test. *P* < 0.05 indicated statistical significance, *P* < 0.01 indicated remarkable statistical significance, and *P* < 0.001 indicated extremely remarkable statistical significance.

## Additional files

**Additional file 1: Figure S1.** Phylogenetic analysis of *Po*Set2 orthologs.

**Additional file 2: Figure S2.** Saturation analysis of the depth of sequencing data. X-axis showed the number of clean reads, whose extreme value was the current volume of sequencing. Y-axis showed the ratio of identified gene numbers to total gene numbers reported in database. (a) Three biological replicates of WT cultivated in a glucose medium. (b) Three biological replicates of Δ*Poset2* cultivated in a glucose medium. (c) Three biological replicates of WT cultivated in cellulose medium. (d) Three biological replicates of Δ*Poset2* cultivated in cellulose medium.

**Additional file 3: Spreadsheet S1.** A total of 469 genes exhibited significant differences (fold change ≥ 2, probability ≥ 0.8) in Δ*Poset2* compared with that of WT in the glucose medium.

**Additional file 4: Spreadsheet S2.** A total of 1457 genes exhibited significant differences (fold change ≥ 2, probability ≥ 0.8) in Δ*Poset2* compared with that of WT in cellulose medium.

**Additional file 5: Table S1.** List of upregulated genes (≥ twofold, FDR < 0.05) in Δ*Poset2* compared with WT with significantly enriched GO terms (GO category: molecular function) in cellulose medium.

**Additional file 6: Figure S3.** Verification of *Po*Swd2 extracted in TAP-MS experiments via Western blot analysis and silver staining. (a) Western blot analysis of affinity-purified tagged *Po*Swd2. (b) Silver staining of TAP-tagged proteins together with associated proteins after one-step (anti-FLAG) affinity purification. (c) Silver staining of TAP-tagged proteins together with associated proteins after two-steps (anti-FLAG and then anti-HA) affinity purification.

**Additional file 7: Spreadsheet S3.** Putative proteins interacting with *Po*Swd2 identified using TAP-MS experiments.

**Additional file 8: Figure S4.** Strategy and results of PCR and Southern blot analysis for the verification of multiple mutants. (a) Strategy and results of PCR for the verification of deletion strain. (b) Strategy and results of PCR for the verification of re-complement and overexpression strains. (c) Strategy and results of Southern blot analysis for the verification of *Poset2* deletion strain. (d) Strategy and results of Southern blot analysis for the verification of *Poset2* overexpression strain.

**Additional file 9: Table S2.** Primers used in this study.

## Data Availability

Whole Genome Shotgun projects were deposited in DDBJ/EMBL/GenBank under the accession number AGIH00000000.1 (https://www.ncbi.nlm.nih.gov/nuccore/AGIH00000000.1). The raw data of expression profiling sequencing were deposited in the NCBI’s Gene Expression Omnibus database under the accession number GSE106558 (https://www.ncbi.nlm.nih.gov/geo/query/acc.cgi?acc=GSE106558). All other data that support the findings of this study can be found in Additional files 1–9.

## Abbreviations

WT: wild type
TFs: transcription factors
pNPC: 4-nitrophenyl β-d-cellobioside
CMC: carboxymethylcellulose
FPKM: fragments per kilobase transcriptome per million mapped reads
GO: gene ontology
COMPASS: complex associated with Set1
qRT-PCR: quantitative real-time reverse transcription PCR
ChIP-qPCR: chromatin immunoprecipitation-quantitative PCR
IP: immunoprecipitation
PMSF: phenylmethanesulfonyl fluoride
Ct: threshold cycle of PCR
