# Supplementary figures and images for: Normal transcription of cellulolytic enzyme genes relies on the balance between the methylation of H3K36 and H3K4 in Penicillium oxalicum
Source: Biotechnol Biofuels. 2019 Aug 20;12:198. doi: 10.1186/s13068-019-1539-z (PMC6700826; doi:10.1186/s13068-019-1539-z)

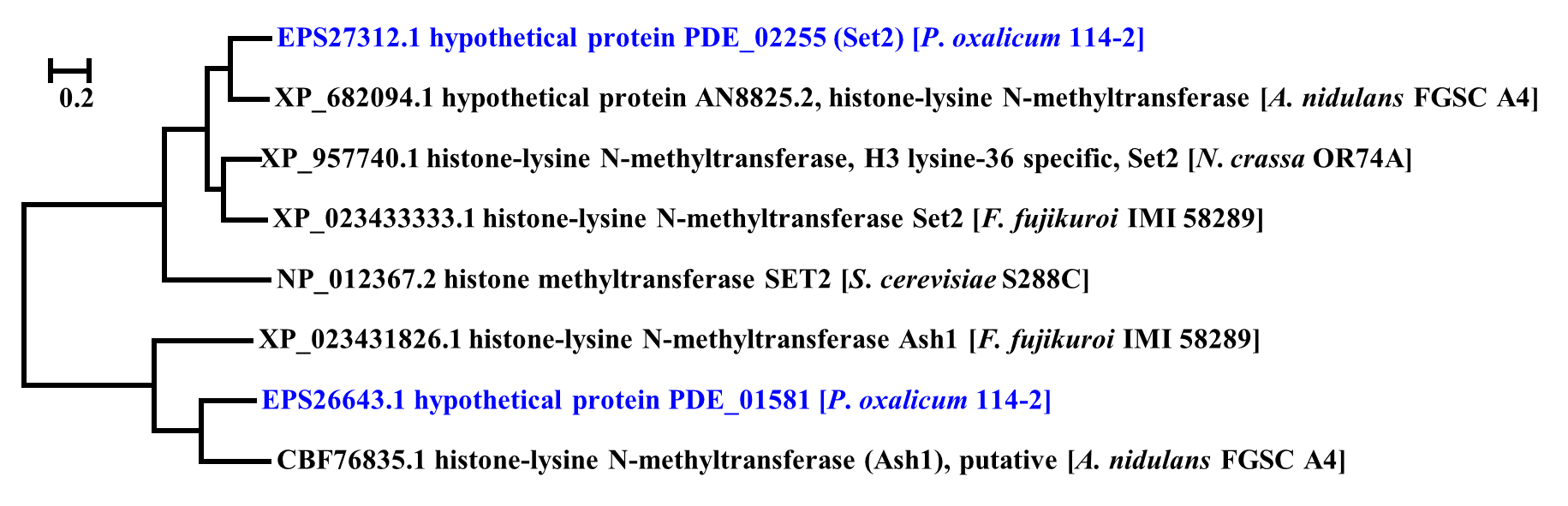

Supplement: Supplementary file 1 — Additional file 1: Figure S1. Phylogenetic analysis of PoSet2 orthologs. [file 13068_2019_1539_MOESM1_ESM.tif]

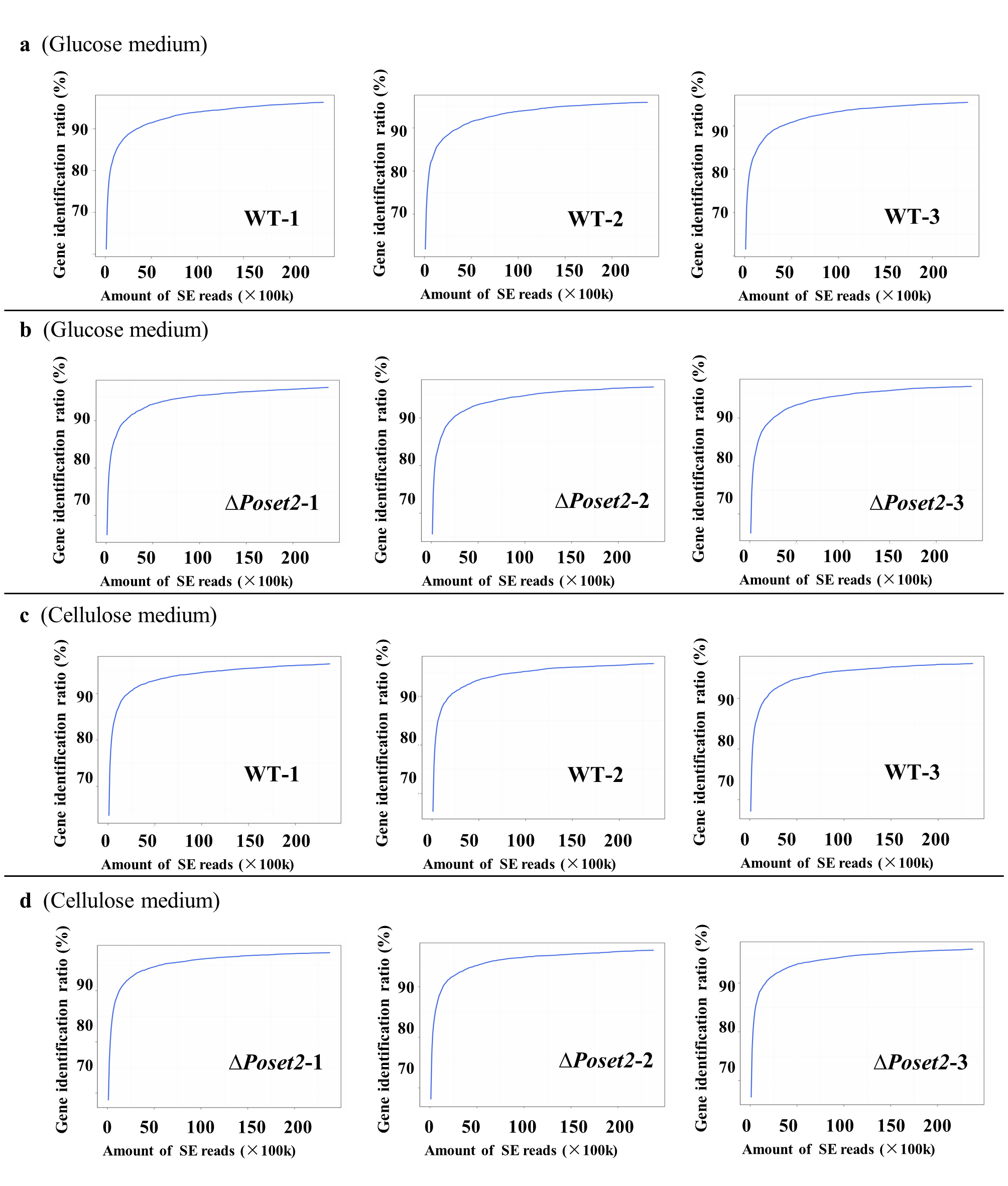

Supplement: Supplementary file 2 — Additional file 2: Figure S2. Saturation analysis of the depth of sequencing data. X-axis showed the number of clean reads, whose extreme value was the current volume of sequencing. Y-axis showed the ratio of identified gene numbers to total gene numbers reported in database. (a) Three biological replicates of WT cultivated in a glucose medium. (b) Three biological replicates of ΔPoset2 cultivated in a glucose medium. (c) Three biological replicates of WT cultivated in cellulose medium. (d) Three biological replicates of ΔPoset2 cultivated in cellulose medium. [file 13068_2019_1539_MOESM2_ESM.tif]

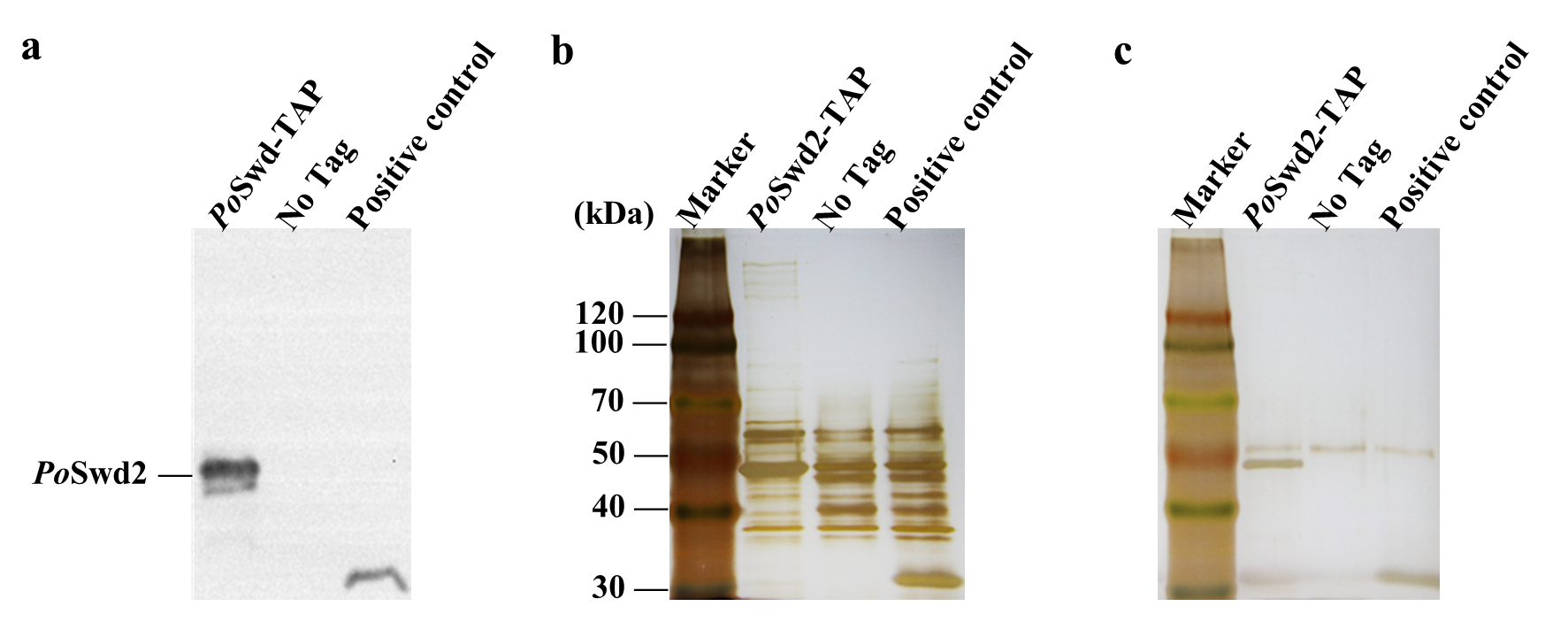

Supplement: Supplementary file 6 — Additional file 6: Figure S3. Verification of PoSwd2 extracted in TAP-MS experiments via Western blot analysis and silver staining. (a) Western blot analysis of affinity-purified tagged PoSwd2. (b) Silver staining of TAP-tagged proteins together with associated proteins after one-step (anti-FLAG) affinity purification. (c) Silver staining of TAP-tagged proteins together with associated proteins after two-steps (anti-FLAG and then anti-HA) affinity purification. [file 13068_2019_1539_MOESM6_ESM.tif]

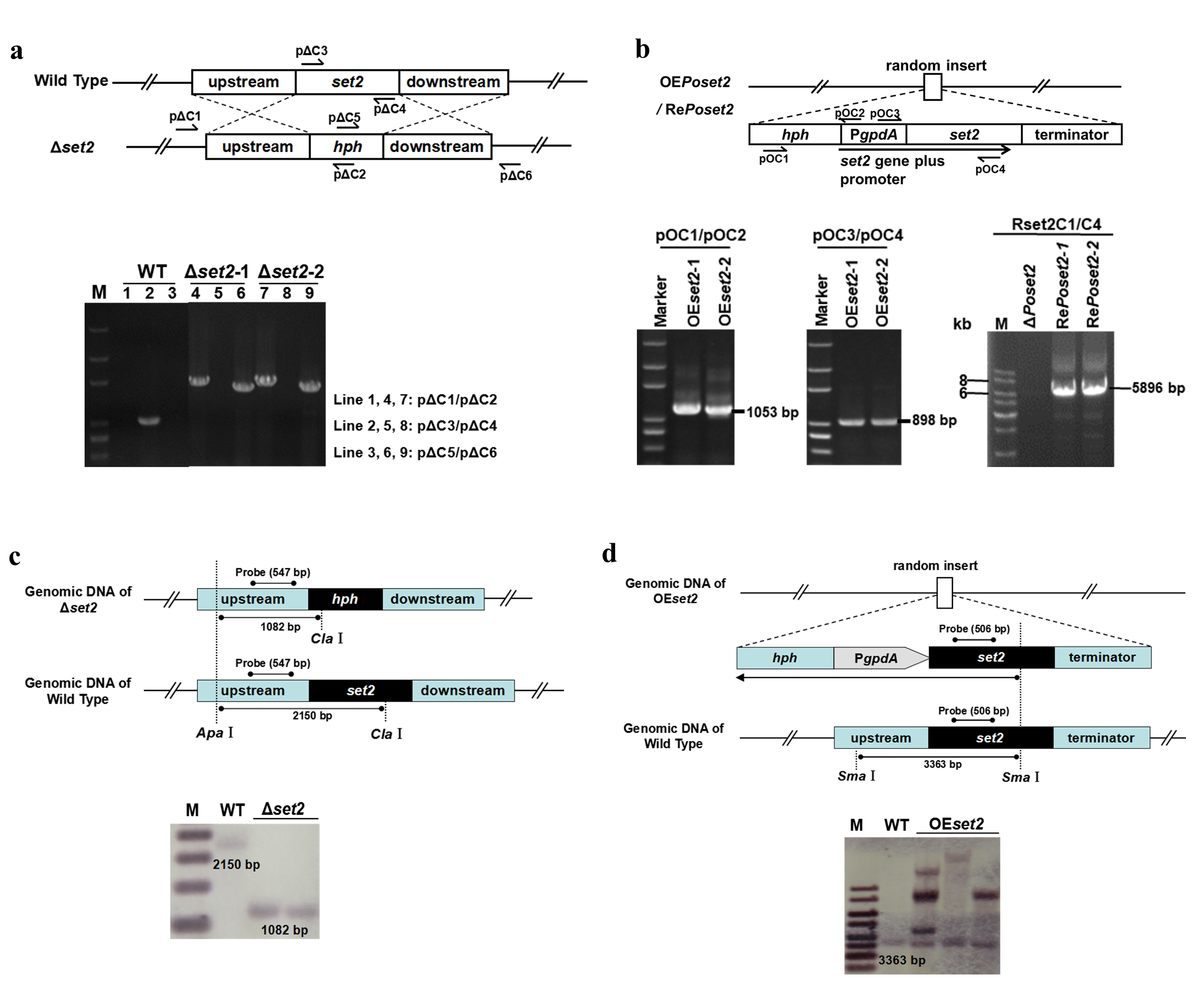

Supplement: Supplementary file 8 — Additional file 8: Figure S4. Strategy and results of PCR and Southern blot analysis for the verification of multiple mutants. (a) Strategy and results of PCR for the verification of deletion strain. (b) Strategy and results of PCR for the verification of re-complement and overexpression strains. (c) Strategy and results of Southern blot analysis for the verification of Poset2 deletion strain. (d) Strategy and results of Southern blot analysis for the verification of Poset2 overexpression strain. [file 13068_2019_1539_MOESM8_ESM.tif]
