# Supplementary material for: Normal transcription of cellulolytic enzyme genes relies on the balance between the methylation of H3K36 and H3K4 in Penicillium oxalicum
Source: Biotechnol Biofuels. 2019 Aug 20;12:198. doi: 10.1186/s13068-019-1539-z (PMC6700826; doi:10.1186/s13068-019-1539-z)
Supplement: Supplementary file 5 — Additional file 5: Table S1. List of upregulated genes (≥ twofold, FDR < 0.05) in ΔPoset2 compared with WT with significantly enriched GO terms (GO category: molecular function) in cellulose medium. [file 13068_2019_1539_MOESM5_ESM.docx]

**Table S1.** List of upregulated genes (≥ 2-fold, FDR < 0.05) in Δ*Poset2* compared with WT with significantly enriched GO terms (GO category: molecular function) when cultivated for 24 h on the condition of cellulose medium.

| **GO-ID** | **Term** | **Gene ID**  **(locus_tag)** | **Description of putative *P. oxalicum* ORF** |
| --- | --- | --- | --- |
| GO:0030248 | Cellulose binding | PDE_00014 | Exoglucanase 1 |
|  |  | PDE_00015 | Putative beta-xylosidase |
|  |  | PDE_00016 | Alpha-L-arabinofuranosidase axhA-2 |
|  |  | PDE_01302 | Endoglucanase E |
|  |  | PDE_02101 | Probable endo-1,4-beta-xylanase B |
|  |  | PDE_02102 | Expansin-B1 |
|  |  | PDE_02514 | Alpha-galactosidase 6 |
|  |  | PDE_02682 | Endo-1,4-beta-xylanase A |
|  |  | PDE_06067 | Arabinoxylan arabinofuranohydrolase |
|  |  | PDE_06438 | Probable mannan endo-1,4-beta-mannosidase E |
|  |  | PDE_07124 | Probable 1,4-beta-D-glucan cellobiohydrolase C |
|  |  | PDE_09226 | Probable endo-beta-1,4-glucanase B |
|  |  | PDE_09278 | Acetylxylan esterase A |
| GO:0004190 | Aspartic-type endopeptidase activity | PDE_01021 | Probable aspartic-type endopeptidase opsB |
|  |  | PDE_04768 | Vacuolar protease A |
|  |  | PDE_07344 | Aspartic protease penicillopepsin |
|  |  | PDE_07927 | Aspartic protease penicillopepsin, PepB |
|  |  | PDE_07930 | Probable aspartic-type endopeptidase opsB |
|  |  | PDE_07933 | Aspergillopepsin-2 |
|  |  | PDE_08305 | Aspergillopepsin A-like aspartic endopeptidase |
|  |  | PDE_09250 | Aubfamily A1A non-peptidase homologues |
| GO:0008810 | Cellulase activity | PDE_02886 | Xyloglucan-specific endo-beta-1,4-glucanase A |
|  |  | PDE_05193 | Probable endo-beta-1,4-glucanase B |
|  |  | PDE_06438 | Probable mannan endo-1,4-beta-mannosidase E |
|  |  | PDE_06439 | Endoglucanase-1 |
|  |  | PDE_07124 | Probable 1,4-beta-D-glucan cellobiohydrolase C |
|  |  | PDE_09014 | Xyloglucan-specific endo-beta-1,4-glucanase A |
|  |  | PDE_09226 | Probable endo-beta-1,4-glucanase B |
| GO:0046933 | Proton-transporting ATP synthase activity, rotational mechanism | PDE_01144 | ATP synthase subunit alpha, mitochondrial |
|  |  | PDE_02994 | ATP synthase subunit gamma, mitochondrial |
|  |  | PDE_03014 | ATP synthase subunit delta, mitochondrial |
|  |  | PDE_04833 | V-type proton ATPase 16 kDa proteolipid subunit |
|  |  | PDE_06476 | ATP synthase subunit alpha, mitochondrial |
|  |  | PDE_06934 | ATP synthase subunit f, mitochondrial |
|  |  | PDE_07279 | ATP synthase subunit beta, mitochondrial |
| GO:0031176 | Endo-1,4-beta-xylanase activity | PDE_00752 | Endo-1,4-beta-xylanase |
|  |  | PDE_02101 | Probable endo-1,4-beta-xylanase B |
|  |  | PDE_02682 | Endo-1,4-beta-xylanase A |
|  |  | PDE_04478 | Endo-1,4-beta-xylanase 6 |
|  |  | PDE_09478 | Endo-1,4-beta-xylanase A |
| GO:0046556 | Alpha-L-arabinofuranosidase activity | PDE_00016 | Alpha-L-arabinofuranosidase axhA-2 |
|  |  | PDE_06067 | Arabinoxylan arabinofuranohydrolase |
|  |  | PDE_07897 | Probable alpha-L-arabinofuranosidase axhA |
|  |  | PDE_09988 | Probable alpha-N-arabinofuranosidase B |
| GO:0046961 | Proton-transporting ATPase activity, rotational mechanism | PDE_01144 | ATP synthase subunit alpha, mitochondrial |
|  |  | PDE_02994 | ATP synthase subunit gamma, mitochondrial |
|  |  | PDE_03014 | ATP synthase subunit delta, mitochondrial |
|  |  | PDE_06476 | ATP synthase subunit alpha, mitochondrial |
|  |  | PDE_07279 | ATP synthase subunit beta, mitochondrial |
| GO:0015932 | Nucleobase-containing compound transmembrane transporter activity | PDE_02295 | GDP-mannose transporter |
|  |  | PDE_08150 | Solute carrier family 28 member 3 |
|  |  | PDE_08167 | UDP-galactose transporter |
| GO:0008121 | Ubiquinol-cytochrome-c reductase activity | PDE_00191 | Cytochrome b-c1 complex subunit 8 |
|  |  | PDE_00284 | Mitochondrial-processing peptidase subunit beta |
|  |  | PDE_07286 | Cytochrome b-c1 complex subunit Rieske, mitochondrial |
|  |  | PDE_09131 | Cytochrome b-c1 complex subunit 2, mitochondrial |
