# Supplementary material for: Normal transcription of cellulolytic enzyme genes relies on the balance between the methylation of H3K36 and H3K4 in Penicillium oxalicum
Source: Biotechnol Biofuels. 2019 Aug 20;12:198. doi: 10.1186/s13068-019-1539-z (PMC6700826; doi:10.1186/s13068-019-1539-z)
Supplement: Supplementary file 9 — Additional file 9: Table S2. Primers used in this study. [file 13068_2019_1539_MOESM9_ESM.docx]

**Table S2. Primers used in the study.**

| Primer name | Sequence (5'-3') |
| --- | --- |
| **Primers for the construction of deletion mutants Δ*Poset2*, Δ*Poset1* and Δ*Poset1*Δ*Poset2*** | |
| pΔset2-UF | TATGGCCATGATTGTATAAGTGATC |
| pΔset2-UR | CTCCTTCAATATCAGTTAACGTCGGATGGTCCAAGTGCACAGCTTCATTC |
| pΔset2-DF | CCGTCACCAGCCCTGGGTTGCTCTATGAGTAACCGATCAAGCAAGC |
| pΔset2-DR | GACCTCTATCCGGGATCAAGATGAAC |
| pΔset1-UF | CGAGACCTCCACCTTCACGCATACC |
| pΔset1-UR | CTCCTTCAATATCAGTTAACGTCGCGTGGAAGGGTCGATTAATTCAACG |
| pΔset1-DF | CCGTCACCAGCCCTGGGTTGCGGTCTCCCTTTATCAATTCCGACCG |
| pΔset1-DR | CATCGATTGGGAGAGTCTACGAATC |
| pΔset2p-UF | GCATCATACACGCGTGCGTGGATAG |
| pΔset2p-UR | GATCCCGTAATCAATTGCCCGACGAACACGGACCGTGTTGAGCTG |
| pΔset2p-DF | GCGGCTCATCGTCACCCCATGCAAGAACGGCTCTATGAGTAACCG |
| pΔset2p-DR | GTGAACGACTGGTTGGCCGTGACAG |
| phph-F | CGACGTTAACTGATATTGAAGGAG |
| phph-R | CAACCCAGGGCTGGTGACGG |
| ptrA-F | GGGCAATTGATTACGGGATC |
| ptrA-R | ATGGGGTGACGATGAGCCGC |
| PΔset2-NF | GCACAATTAGGTTCCTCGTGGACTG |
| pΔset2-NR | GGTGCCTCTCGTGGCTGGCTCCGTG |
| PΔset1-NF | CAAGCATTGTACCTGTCCAGTGGCG |
| pΔset1-NR | CTGACATCTTCCCTCTGATAGCTTG |
| PΔset2p-NF | GTGATAACTCGGATGGTTCATGATC |
| pΔset2p-NR | CACGACAGCCACCGAGCTGAGTGTC |
| **Primers for the verification of deletion mutants** | |
| pΔset2C1 | ATAGGCTACTGATGCTTCACAGGTC |
| pΔset2C2 | CTAAATGAACCATCTTGTCAAACGAC |
| pΔset2pC2 | CTCCTAGCTTTTACATTTCGTTAC |
| pΔset2C3 | CGCTGTCGGAACGGAAGACGTCAAC |
| pΔset2C4 | GATTGCCCATGTACACCCACCGCCTC |
| pΔset2C5 | GAGACTGAGGAATCCGCTCTTGGCTC |
| pΔset2pC5 | GAACTCGTTAGCTCGTAATCCAC |
| pΔset2C6 | CAGTATCGCCGACGTCCTCGGTGTC |
| pΔset1C1 | GCGCATGGTTGGGTTGGAAACTATG |
| pΔset1C2 | CTAAATGAACCATCTTGTCAAACGAC |
| pΔset1C3 | GTGTTTGACAGGTTTGCGCTGCGAC |
| pΔset1C4 | CTTAAATCCACGGTATCCCAACCATG |
| pΔset1C5 | GAGACTGAGGAATCCGCTCTTGGCTC |
| pΔset1C6 | CGAGCAATTAGAAGTGGACCTTCAG |
| **Primers for the construction of Re*Poset2* and Re*Poset1*** | |
| ptrA-F | GGGCAATTGATTACGGGATC |
| ptrA-R | ATGGGGTGACGATGAGCCGC |
| pReset2-F | GCGGCTCATCGTCACCCCATCTCGGATGGTTCATGATCACTGGAAC |
| pReset2-R | CAATGCTCTACATCTAGGGTAGTTTG |
| pReset2-NF | GCTCACCGGTCAAAGCTAAAGAGGAG |
| pReset2-NR | GCAGAGTTGATTGTCGCAACACTTTG |
| pReset1-F | GTTGGCAAATGTCTAACTTGCTGGAG |
| pReset1-R | GATCCCGTAATCAATTGCCCCTTATGGTTGCTTATACCCTAGACG |
| pReset1-NF | GATCAGGTACCATGACCGCAGTTTG |
| pReset1-NR | ATGGGGTGACGATGAGCCGC |
| **Primers for the verification of Re*Poset2* and Re*Poset1*** | |
| pRset2C1 | GTAAGGTCGTGGTTTCTGAGACCGTC |
| pRset2C2 | CGACGTGTCGGGATCTTCTGTCTC |
| pRset2C3 | CTATCAACGCTCCTGTCATCATCAG |
| pRset2C4 | GACTGATGATTCGTGTTTGAGAGATG |
| pRset1C1 | GTTGACCTGGATCGAGATGGAATTG |
| pRset1C2 | CTCCTAGCTTTTACATTTCGTTAC |
| **Primers for the construction of overexpression mutant OE*Poset2*** | |
| phph-F | CGACGTTAACTGATATTGAAGGAG |
| phph-R | CAACCCAGGGCTGGTGACGG |
| pgpdA-F | CCGTCACCAGCCCTGGGTTGAGTCAGACGGCGTAACCAAAAGTC |
| pgpdA-R | GGTGATGTCTGCTCAAGCGG |
| pOEset2-F | CCGCTTGAGCAGACATCACCATGTCCGCTCATGGCAACGCGGAC |
| pOEset2-R | CAAAGACGGCTCCTCTGAAGAAGAAAG |
| pOEset2-NF | GTCGTTTGACAAGATGGTTCATTTAG |
| pOEset2-NR | CTCTACATCTAGGGTAGTTTGGTTG |
| **Primers for the verification of OE*Poset2*** | |
| pOset2C1 | CAATGGCCGCATAACAGCGGTCATTG |
| pOset2C2 | CGTTGTAGGGGCTGTATTAGGTCTCG |
| pOset2C3 | CGTCGGCGAAATAGCATGCCATTAAC |
| pOset2C4 | GTTGGCCTGGTTCCTCTTTCACGAC |
| **Primers for the construction of eGFP labeled strains *Po*Set2-GFP and *Po*Set1-GFP** | |
| pGset2-UF | CTGTTGCTAGTAATCCTCAGACACG |
| pGset2-UR | CAGCTCCTCGCCCTTGCTCACCATTCCTCCTCCTCCTGATTGCCCATGTACACCCACCGCCT |
| pGFP-F | ATGGTGAGCAAGGGCGAGGAGCTG |
| pGFP-R | TTACTTGTACAGCTCGTCCATGCCG |
| pyrG-F | CGGCATGGACGAGCTGTACAAGTAAGCAACTTCCTCGAGAACGCG |
| pyrG-R | CCCTTTTAGTCAATACCGTTAC |
| pGset2-DF | GTAACGGTATTGACTAAAAGGGATGGCTGAACTGAGGCCCGCCTGC |
| pGset2-DR | CAGTATCGCCGACGTCCTCGGTGTC |
| pGset2-NF | GCGGCTTCTGCATGATCAGAGATAC |
| pGset2-NR | GACCTCTATCCGGGATCAAGATGAAC |
| pGset1-UF | GACCCTTCCACGGGTTGCTGGGCCAG |
| pGset1-UR | CAGCTCCTCGCCCTTGCTCACCATTCCTCCTCCTCCGTTGAGGAAACCTTTGCACCCCGTG |
| pGset1-DF | GTAACGGTATTGACTAAAAGGGGCCGTTATGTTACTCCCATCCTTTATC |
| pGset1-DR | CTTTCCATTTCATCCGCTGTCTCCAG |
| pGset1-NF | CCGCCCTTGTGGTCAAGCCTCGTCG |
| pGset1-NR | CTCTCGACGGCCACCCAAGATGAAC |
| **Primers for the verification of *Po*Set2-GFP and *Po*Set1-GFP** | |
| pGset2C1 | GAATGAAGCTGTGCACTTGGACCATC |
| pGset2C2 | ATGAGAAGTTCAACCAGAGATAG |
| pGset1C1 | CGCCAAACTGTACGGCCAAGATCATC |
| pGset1C2 | GGAGCAACAAGAATAAAACCCAGAGG |
| **Primers for the amplification of probes of Southern hybridization** | |
| SPΔset2-F | CTGTTGCTAGTAATCCTCAGACACG |
| SPΔset2-R | GATGGTCCAAGTGCACAGCTTCATTC |
| SPΔset1-F | CAGTGCCAAGTGTATGATTGGTATG |
| SPΔset1-R | GTCGCAGCGCAAACCTGTCAAACAC |
| SPOE-F | CTATGAATGGGCATGAGGAGACCTC |
| SPOE-R | GAACTCATACAGACCGCGCAGCTTG |
| **Primers for the transcription analysis by qRT-PCR** | |
| Actin-QF | GTTCCATTCTCGCCTCCCTCT |
| Actin-QR | AGAAGCACTTGCGGTGAACGA |
| Set2-QF | GCGAAAAGCATCAAAAAACGG |
| Set2-QR | TTTGACTCTGTCGCCGAAGCC |
| BrlA-QF | GTCAGGAACATCTCAAGCGGC |
| BrlA-QR | GCTTGCTGTGGGTCTTGGTG |
| Xyn10A-QF | GGTCTCCAGGCTCACTTCATC |
| Xyn10A-QR | GTCGAGGGCAAGTTCATACG |
| Xyn11A-QF | CCATGACTGTGTCTGCCGGCAG |
| Xyn11A-QR | GAGCCGCTACCAGAGCCAGAAC |
| Abf62A-QF | GGTCGTCATCCCAGTGCCTCTTCAGT |
| Abf62A-QR | GCGATAGATCTTGCCATTGTCTCCCG |
| Cbh1-QF | CCACCACCACTACCAGCAAGG |
| Cbh1-QR | GTAGCCAACACCACCGCACT |
| Cbh2-QF | GAGTGTGACGGTACCTCGGACAC |
| Cbh2-QR | CTTGGGTTGGCGTTGACGAGCAG |
| Eg1-QF | ACCGCTGCTCAGACCACGAC |
| Eg1-QR | TGGGTCCCGAGTAGCCAACG |
| Eg2-QF | CAAGACCACCACTACAGCCGCAC |
| Eg2-QR | CGAGTAGTAGTCGTTCTGCTTC |
| CreA-QF | TGGGTACGAGTGAACTCCATCTT |
| CreA-QR | TGTGACCTTGACCAGGACTGTAA |
| ClrB-QF | AGCACAAGTCGAGATGGGATT |
| ClrB-QR | CGCTTGCTGGCTTCGTAAAT |
| XlnR-QF | CGATCCGCTCTTGCCCAGGTA |
| XlnR-QR | GGGCGAGAACTTCACGTCTG |
| AmyR-QF | TCACCATCGGCAACTTTCTCC |
| AmyR-QR | TCGCCTTCCATGTGAATCTCG |
| **Primers for *abf62A*** | |
| Abf62A-1F | CATGTCTAGCAAAACCCAACCAG |
| Abf62A-1R | CGACCGGACGACTCCACTGTCTG |
| Abf62A-2F | GAGTACTCCCTCAAGCCTCCATC |
| Abf62A-2R | CGAGACCCTGACGTTCATCTGAG |
| Abf62A-3F | GATCTGGAGGTTTGAGTTGCAAG |
| Abf62A-3R | CTCCCTCTCATCCGTTGCAATTG |
| Abf62A-4F | GCCTGCACCATTGGTCCAGAAGG |
| Abf62A-4R | CAAGCACACGCAGGTCAACCAG |
| Abf62A-5F | CCGAGGTTCATACCGTGCGAAG |
| Abf62A-5R | CGCATCTCCACATGCCATTATC |
| Abf62A-6F | CACTGGGAGTACCATTGGTTAG |
| Abf62A-6R | CATGTCTAGCAAAACCCAACCAG |
| **Primers for *xyn11A*** | |
| Xyn11A-1F | GACCCTGTTGCCTCCACAATTTC |
| Xyn11A-1R | CATGTCTAGCAAAACCCAACCAG |
| Xyn11A-2F | CGACCGGACGACTCCACTGTCTG |
| Xyn11A-2R | GAGTACTCCCTCAAGCCTCCATC |
| Xyn11A-3F | CGAGACCCTGACGTTCATCTGAG |
| Xyn11A-3R | GATCTGGAGGTTTGAGTTGCAAG |
| Xyn11A-4F | CTCCCTCTCATCCGTTGCAATTG |
| Xyn11A-4R | GCCTGCACCATTGGTCCAGAAGG |
| Xyn11A-5F | CAAGCACACGCAGGTCAACCAG |
| Xyn11A-5R | CCGAGGTTCATACCGTGCGAAG |
| Xyn11A-6F | CGCATCTCCACATGCCATTATC |
| Xyn11A-6R | CACTGGGAGTACCATTGGTTAG |
| **Primers for c*el6A / cbh2*** | |
| Cbh2-1F | CGAGACCCTTTGACATCTGCAAC |
| Cbh2-1R | GCAACTTCTGCTCCGCTTCGTC |
| Cbh2-2F | GTGTATGCGGGCGTCCTGGGGTAC |
| Cbh2-2R | GAGGGTTGGAGAGTGAAGAATC |
| Cbh2-3F | GGCTGTCGGAATGAAGCCTAAG |
| Cbh2-3R | GTGTCTATCCTTGATTGTCAATG |
| Cbh2-4F | CAGAGAACATCAGCTTGGGCAC |
| Cbh2-4R | CACCACCTGGAAGAATCAAGATG |
| Cbh2-5F | CACCCATTGCCGGTATCTTTGTC |
| Cbh2-5R | CAGGGTACTTGAGAAGCTGAGCG |
| Cbh2-6F | GAGTGTGACGGTACCTCGGACAC |
| Cbh2-6R | CTTGGGTTGGCGTTGACGAGCAG |
| **Primers for *cel5B / eg2*** | |
| Eg2-1F | GTGGAACACTTCGCCTTCTCGC |
| Eg2-1R | GTGACTGCGTCTGCCAGAAGAG |
| Eg2-2F | CACGTGTGACTTATTCAGCGATG |
| Eg2-2R | GCGTGGGTCCATGTATCAGGTC |
| Eg2-3F | GAGGATCTGCTCGGTTGAATCTC |
| Eg2-3R | GAAATCCAATGTATGCAGACTCG |
| Eg2-4F | GAGATACGATTTGATTCTTGC |
| Eg2-4R | GTTCACGCCCATCAGCGATTAG |
| Eg2-5F | GATGAATCTCCTGTTTCTGACC |
| Eg2-5R | CAGTCCACGAGTTTCCTTCAAC |
| Eg2-6F | CAAGACCACCACTACAGCCGCAC |
| Eg2-6R | CGAGTAGTAGTCGTTCTGCTTC |
| **Primers for *swd2*** | |
| Swd2-1F | GCACCACAAAGACATCAGATCCTAG |
| Swd2-1R | GATGCATCTCTGGAGCCTTCTCAGC |
| Swd2-2F | GCAACAGGGCACCACTCCACCTCTC |
| Swd2-2R | GAGCAGCGTCGCGGTTTCCTCTTTC |
| Swd2-3F | GCTGGCGCGTCCTGAGCTTTCCCTC |
| Swd2-3R | GAAACTAGATCCGAGATCCGAATAG |
| Swd2-4F | CATGGCCGAAGGCCCGCAGCTTACG |
| Swd2-4R | GTGCTGTGAGGTTGAATGGTTATGC |
| Swd2-5F | GTCTCCAGGTAGCGATGCCTTCATC |
| Swd2-5R | CCGAAGCAGACGGGTCAAATGCAAC |
| Swd2-6F | GACAAGCGAACGTATCACTCCAAC |
| Swd2-6R | GTCGGAGGGTCTGGATGGCTCATC |
| **Primers for *swd3*** | |
| Swd3-1F | CGTCTGGTCACGAATACTCTGGTAG |
| Swd3-1R | CATCTCCAAGTTCCGTCATGAACGTC |
| Swd3-2F | GAGTCCTCGGCTTCACTTGACCTCG |
| Swd3-2R | GTGAGTGTGTGACTTTACATCACCG |
| Swd3-3F | GATATGGGCGATAAGTTTATCGCTTG |
| Swd3-3R | GCAATTAAATTGTCGAAGACGGCGAC |
| Swd3-4F | GATAATGCGCGCCCCCGAACTCGAC |
| Swd3-4R | CTCCTGTTTCTGGACCAGCTTCGTC |
| Swd3-5F | CTGTAAGCTCGTGCCGACCTATATC |
| Swd3-5R | GGAGTGTGCACGACCCTAGAGCGTG |
| Swd3-6F | GACGGCCGTCACTGTTGGCGTCAGC |
| Swd3-6R | CATTACCACATCTTCGGTGTGGGTC |
| **Primers for *set1*** | |
| Set1-1F | CAAGAGGTGAAACTCTTCCGTGAGG |
| Set1-1R | GGACTCTCATTGACTACAGCAGTTC |
| Set1-2F | GATCGCTCCTACCGCCTGCCTGTTC |
| Set1-2R | GTCTGGAAAACGGGACAGCAGCAGC |
| Set1-3F | GCCCTTGTGGTCAAGCCTCGTCGCC |
| Set1-3R | GAGATATGGCTACACGCCCGGGACG |
| Set1-4F | CGCAGAAGCGTGGCAAGGTTAGAG |
| Set1-4R | CAGCCGCAGCGCCATTGAATGACG |
| Set1-5F | CATCGCTGCGCCAACCTTGTACGAC |
| Set1-5R | GAGTGCCCACTGAACTACTGACTCC |
| Set1-6F | GATCACGACCCGAGCAGTCTCCTTTC |
| Set1-6R | GCTATCCCATTCGCGTTCAAACTTG |
